# Chromatin-based, *in cis* and *in trans* regulatory rewiring underpins distinct oncogenic transcriptomes in multiple myeloma

**DOI:** 10.1038/s41467-021-25704-2

**Published:** 2021-09-14

**Authors:** Jaime Alvarez-Benayas, Nikolaos Trasanidis, Alexia Katsarou, Kanagaraju Ponnusamy, Aristeidis Chaidos, Philippa C. May, Xiaolin Xiao, Marco Bua, Maria Atta, Irene A. G. Roberts, Holger W. Auner, Evdoxia Hatjiharissi, Maria Papaioannou, Valentina S. Caputo, Ian M. Sudbery, Anastasios Karadimitris

**Affiliations:** 1 Department of Molecular Biology and Biotechnology, University of Sheffield, Sheffield, UK; 2 Hugh & Josseline Langmuir Centre for Myeloma Research, Centre for Haematology, Department of Immunology and Inflammation, Imperial College London, London, UK; 3 Department of Haematology, Hammersmith Hospital, Imperial College Healthcare NHS Foundation Trust, London, UK; 4 MRC Molecular Haematology Unit and Paediatrics, MRC Weatherall Institute of Molecular Medicine, Oxford, UK; 5 First Department of Internal Medicine, Division of Haematology, AHEPA University Hospital, Aristotle University of Thessaloniki, Thessaloniki, Greece

**Keywords:** Gene regulatory networks, Systems biology, Myeloma

## Abstract

Multiple myeloma is a genetically heterogeneous cancer of the bone marrow plasma cells (PC). Distinct myeloma transcriptome profiles are primarily driven by myeloma initiating events (MIE) and converge into a mutually exclusive overexpression of the *CCND1* and *CCND2* oncogenes. Here, with reference to their normal counterparts, we find that myeloma PC enhanced chromatin accessibility combined with paired transcriptome profiling can classify MIE-defined genetic subgroups. Across and within different MM genetic subgroups, we ascribe regulation of genes and pathways critical for myeloma biology to unique or shared, developmentally activated or de novo formed candidate enhancers. Such enhancers co-opt recruitment of existing transcription factors, which although not transcriptionally deregulated per se, organise aberrant gene regulatory networks that help identify myeloma cell dependencies with prognostic impact. Finally, we identify and validate the critical super-enhancer that regulates ectopic expression of *CCND2* in a subset of patients with MM and in chronic lymphocytic leukemia.

## Abstract (web summary)

Despite extensive genetic heterogeneity, nearly half of all multiple myeloma (MM) cases are driven by cyclin D2 (CCND2) over-expression. Here the authors dissect the chromatin landscape of MM to provide insights into the transcriptional regulatory landscape driving MM and divergent transcriptomes corresponding to different MM genetic subtypes.

## Introduction

Multiple myeloma (MM) is a common, genetically heterogeneous and incurable cancer of the bone marrow (BM) plasma cells (PC)^1^, the terminally differentiated, immunoglobulin-secreting B lineage cells. The first level of genetic heterogeneity in MM is imparted by well-defined myeloma-initiating events (MIE) that are associated with distinct transcriptome profiles. In nearly half of MM cases, MIE include overexpression of the oncogenes *CCND1*, *MAF* and *MMSET* by their juxtaposition to the immunoglobulin heavy chain (*IgH*) enhancer, thus defining the t(11;14), t(14;16) and t(4;14) cytogenetic subgroups, respectively. Hyperdiploidy (HD), a functionally heterogeneous subgroup characterised by additional odd number chromosomes, is the MIE in the rest of MM cases^2^. Secondary events comprising copy number aberrations, single nucleotide variants and indels generate additional genetic heterogeneity and further shape the distinct impact of the MIE on oncogenic transcriptomes^3^. This heterogeneity converges, in most cases, to a functionally dichotomous, mutually exclusive overexpression of the cell cycle regulators CCND1 and CCND2 to which myeloma PC remain addicted, irrespective of primary or secondary genetic events^4,5^. The transcriptional mechanisms that result in *CCND2* over-expression, seen in nearly 50% of MM cases and spanning all genetic subgroups except t(11;14), are not fully known. A previous study of a single myeloma cell line identified but not validated a super-enhancer that spans the promoter of *CCND2*, leaving the possibility of a distal enhancer/super-enhancer regulating transcription of *CCND2* unexplored^6^.

Chromatin accessibility profiling by ATAC-seq has been used to characterise the regulatory landscape of hundreds of different solid tumour and blood cancers, such as chronic lymphocytic leukaemia (CLL)^7,8^. Further, by means of transcription factor (TF) footprinting, ATAC-seq allows inference of TF binding profiles. This, in combination with paired transcriptome profiles, enables the construction of gene regulatory networks^8,9^. Such networks may identify TFs with previously unrecognised roles in the biology of a given cancer.

In previous studies^10,11^ of chromatin-based regulatory changes of gene expression, myeloma was treated as a homogeneous cancer. However, how heterogeneity links chromatin accessibility and regulatory status with distinct oncogenic gene expression profiles has not been elucidated, while global insights into the interplay between *in cis* and *in trans* regulatory factors of gene transcription in MM are limited.

Here, by integrating chromatin accessibility dynamics of myeloma PC with their respective transcriptome profiles and other epigenetic datasets we resolve the chromatin changes that regulate distinct oncogenic transcriptomes and biological pathways. Through this process we discover *cis-* and *trans-*regulators of myeloma biology, including those involved in the regulation and aberrant expression of *CCND2* in MM.

## Results

### Enhanced accessibility of distal chromatin elements is associated with gene over-expression in myeloma plasma cells

**Fig. 1:**
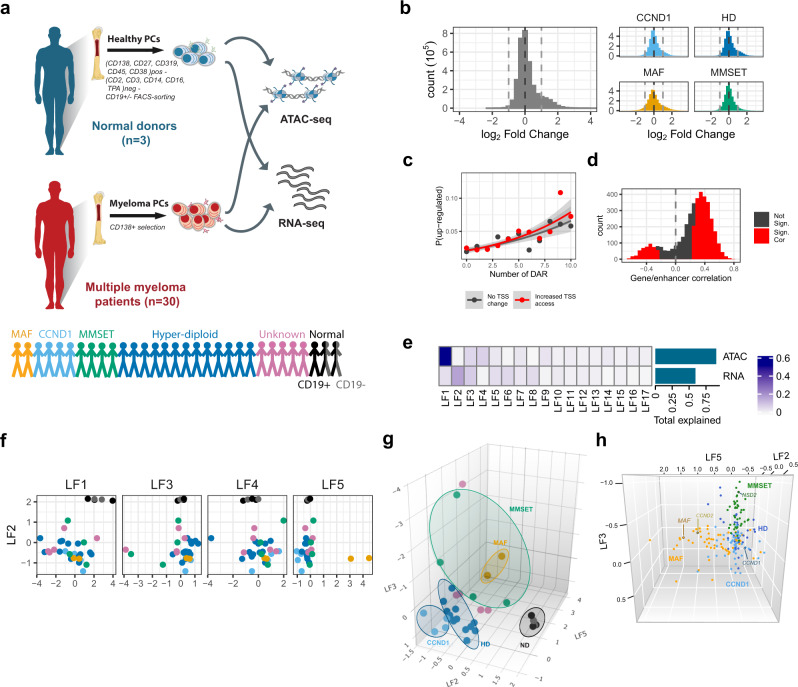
Over-accessible distal chromatin classifies myeloma genetic subgroups. **a** Study patient population and design. Transcriptome and chromatin accessibility were assessed by RNA-seq and ATAC-seq respectively in myeloma PC from 30 MM patients and PC from three healthy donors. For two of the controls we obtained samples of both CD19^+ ^and CD19^−^ PC. **b** Changes in average ATAC-seq signal over pan-myeloma (left) and subgroup (right) peaks expressed as normalized log_2_ myeloma/normal PC read count. Significant changes in the pan-myleoma plot (left) are shown in a darker colour. **c** Logistic regression of number of differentially accessible regions (DAR) within 500 kb of a gene (*x*-axis), whether a gene has a significantly more open promoter in myeloma (red – yes, black – no) on the probability of the gene being differentially upregulated in myeloma. Points represent fraction of genes upregulated with a given number of DAR within 500 kb and TSS status, and lines represent model fit, grey ribbon 95% confidence interval. Genes with 10 or more DAR were pooled together. **d** Correlation of signal for differentially accessible ATAC-seq regions and differentially expressed genes within the same topology associating domain (TAD). **e** Variance explained by the first 17 latent factors (LF) for each of RNA-seq and ATAC-seq signals as calculated by multi-omics factor analysis (MOFA). **f**, **g** LF scores for each sample for the first five latent factors in the MOFA model. Subtype for each sample is denoted by colour, as per (**a**). LF1 and LF2 distinguish normal from MM samples, LF5 distinguishes MAF and CCND1 samples from the rest. **h** LF scores for a subset of regions and genes used as predictors by MOFA analysis and previously identified as MM-subgroup classifiers^12^. The main driving oncogenes *MAF, CCND1, CCND2* and *NSD2* are highlighted here.

We isolated fresh, highly purified BM (CD19^+ /−^) PC from three healthy normal donors (ND) and myeloma PC from 30 MM patients, covering the main MIE subgroups as defined by fluorescent in situ hybridization (Fig. 1a, Supplementary Fig. 1a and Supplementary Data 1) and spanning both diagnostic and relapsed stages of the disease. For each sample, we obtained paired chromatin accessibility and transcriptome profiles by ATAC-seq and RNA-seq, respectively.

ATAC-seq analysis generated 295,238 chromatin accessibility peaks from all samples (Supplementary Fig. 1b). These peaks were non-randomly distributed on promoters, coding and intergenic regions, and were found to be unique or shared between two or more genetic subgroups (Supplementary Fig. 1b and c). In each genetic subgroup and overall, chromatin accessibility of myeloma PC was enhanced in comparison to ND PC (Fig. 1b and Supplementary Data 2).

Transcriptome profiling identified 3036 differentially expressed genes (DEG) between myeloma and ND PC, including overexpression of known MIE-driven oncogenes in myeloma PC (Supplementary Fig. 1d and Supplementary Data 3). In addition, using genesets previously identified as classifiers for each myeloma genetic subgroup and the corresponding MIE^12^, we clustered the transcriptome profiles of our samples along with those of 892 myeloma PC samples from the MMRF CoMMpass study (Supplementary Fig. 1e). This analysis confirmed the accuracy of genetic subgroup annotation of our cohort samples by FISH, and assisted in the classification of samples with unknown cytogenetics status.

Malignant vs normal state analysis showed that over-expressed genes were associated with significantly more non-TSS peaks with increased accessibility than non-overexpressed genes (Supplementary Fig. 1f), and the distance from over-expressed genes to the closest such peak was shorter (Supplementary Fig. 1g). Combined logistic regression analysis revealed that the number of more accessible non-TSS regions was predictive of over-expression (*p* < 2 × 10^−16^) but a more open promoter was not (*p* = 0.5; Fig. 1c), although gain in TSS accessibility was mildly predictive when ignoring distal peaks (over expression odds ratio of 1.3 for TSS accessibility gain vs not, *p* = 0.01).

Using the 3D genome architecture of GM12878 B cells as reference^13^, we found a significant enrichment for differentially accessible regions (DARs) correlating with DEG within the same topologically associating domain (TAD)^14^ as compared to permuted DEG-DAR associations (*p* < 0.001, see Fig. 1d). We also found that the strength of correlations, i.e., mean DAR-DEG correlation coefficient and the percentage (%) of DAR-DEGs that show significant correlation (*p* < 0.05), is higher when DEG-DAR pairs are in the same TAD compared to distance matched controls not in the same TAD (*p* = 0.003, Supplementary Fig. 1h and i).

Thus, myelomagenesis leads to an overall increase in chromatin accessibility and, within the spatial limits of TADs, chromatin decondensation at distal regions rather than at promoters is associated with gene over-expression in the cancer state.

### Over-accessible chromatin in myeloma PC partially distinguishes distinct myeloma transcriptomes according to MIE

Next, we sought to explore and potentially resolve the heterogeneity present across MM patients in an unsupervised manner, by employing multi-omics factor analysis (MOFA)^15^ (Fig. 1e and f, Supplementary Fig. 2a). We found that within the combined ATAC-seq/RNA-seq model, chromatin accessibility accounted for more variance than expression (Fig. 1e). Of the top five identified MOFA latent factors (LF), the mostly chromatin accessibility-driven LF1 and the mainly transcriptome-driven LF2 distinguished ND from myeloma PC; the next three factors, LF3-5 separate different MM patients from another. To investigate possible sources of this heterogeneity, we superimposed the MIE-defined genetic subgroup characterisation of each sample. This revealed a clear separation of ND, MAF and CCND1 subgroups and less so of HD and MMSET by combined LF2, 3 and 5 (Fig. 1f and g).

By interrogating further the MOFA predictors underlying the observed segregation, we found that genes, previously identified as MIE-defined genetic subgroup classifiers, along with their linked chromatin changes delimited in the same TAD, displayed the same subgroup-segregation profile on those three LF (Fig. 1h). We validated this using two MAF-, two CCND1- and one MMSET-translocated myeloma cell lines as a test set not used in training the model, and confirmed separation of samples according to MIE in the LF2-LF3-LF5 MOFA space (Supplementary Fig. 2b–d).

To explore the properties of MOFA clustering further, we applied silhouette width (i.e., a measurement of cluster cohesion and separation) and Discriminant Ratio (i.e., the ratio of between group variance to within group variance; see “Methods”). Silhouette width showed that the combination of accessibility and transcriptome information of LF1-5 provides a clear separation of ND, MAF and CCND1 but not HD and MMSET subgroups, and this separation was clearer than in the model built using RNA-seq alone (Supplementary Fig. 2e).

When the discriminant ratio was applied to measure the ability of each LF to discriminate subgroups, LF5 had the highest ratio at 22.7. To understand how much discriminatory power ATAC-seq data was adding to the model, we trained a second model using the transcriptome data only. The best discriminating factor in this model was LF4, with a discriminant ratio of 11.3, showing that the addition of chromatin accessibility data almost doubled the power of the most discriminatory factor to separate subgroups. Applying linear discriminant analysis to each model (Supplementary Fig. 2f), showed the subtypes are well separated in the combined ATAC-RNA model, with the exception of one MMSET sample located with the HD samples, and one HD sample located with the CCND1 samples. Conversely, for the RNA-only samples, while MAF and ND samples are well separated, the HD, MMSET and CCND1 samples are largely overlapping.

Since there was no difference between CD19^+ ^and CD19^−^ ND PC cells, they were merged for subsequent analyses.

Given these findings, we conclude that combined epigenome–transcriptome-based categorisation of MM maps away myeloma PC from normal PC while within the main MM genetic subgroups it clearly delineates the MAF and CCND1 subgroups and less so the HD and MMSET subgroups.

### The myeloma enhanceome is linked to known and novel MIE-associated genes and biological pathways

**Fig. 2:**
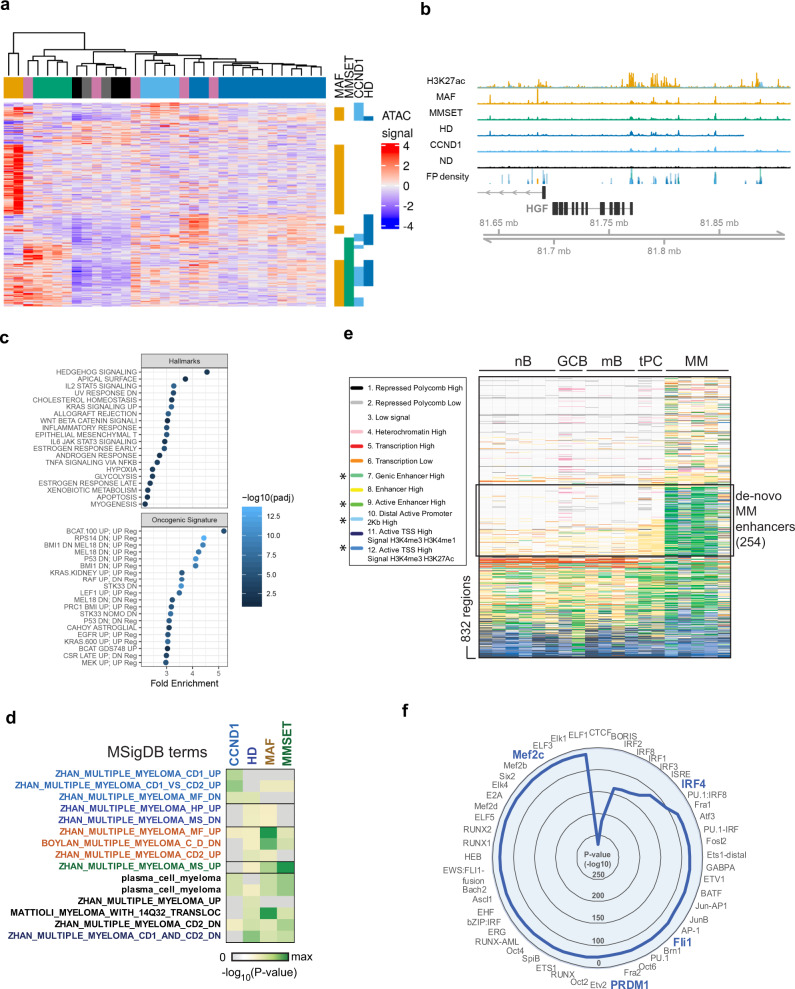
Developmental origins and oncogenic pathways regulated by the myeloma enhanceome. **a** Heatmap showing the ATAC-seq signal for all peaks found to be differentially open and within 1 Mb of a significantly differentially regulated gene. Data are row scaled. Samples are clustered using Pearson’s correlation distance and the bar above the columns shows the subtype of the sample coded as in Fig. 1a, i.e., MAF: orange, CCND1: light blue, MMSET: green, HD: blue, others: pink and ND PC: black/grey. Vertical bars to the right highlight regions where signal is >2-fold different to ND PC. **b** Example region around *HGF* showing: average ATAC-seq signal in each subtype; H3K27ac signal in a *MAF*-translocated cell line (orange) or *CCND1*-translocated cell line (blue); FP density: density of footprints in each of the ATAC-seq signals. **c**, **d** Enrichment analysis for upregulated genes with a peak of increased accessibility within 1 Mb using gene sets from the “Oncogenic signatures”, “Hallmarks” or “Curated gene sets” subsets of the MSigDB database. **e** Chromatin state of differentially open pan-MM peaks across a developmental range of B-cell types as determined by the Blueprint Epigenomics consortium using ChromHMM (nB naive B cells, GCB germinal center B cell, mB memory B cell, tPC tonsil PC, MM myeloma PC). Enhancers with de novo formed peaks in myeloma (254) are indicated. Chromatin states with strong combined H3K27ac and H3K4me1 signals were considered as active enhancers and indicated by an asterisk. **f** Radial plot of Homer motif analysis displaying the top 50 over-represented TF motifs in de novo myeloma enhancers.

To expand our ability to delineate heterogeneity and given the importance of distal genetic elements into the regulation of MM transcriptomes, we next sought to identify the changes that together comprise the myeloma-specific enhanceome using a genetic subgroup supervised approach. We identified distal regions and genes where genetic subgroup (i.e., MAF/MMSET/CCND1/HD/ND) is a significant explanatory variable for accessibility/expression using an omnibus LRT test to maximise power compared to pairwise testing (adjusted LRT *p*-value <0.05 and >2-fold change between at least one MIE and ND, Supplementary Data 3). Integration of these two sets (4635 regions and 3,096 genes) gave 4199 DAR–DEG pairs within 1 Mb of one another (Supplementary Data 4), comprising 2581 unique DAR and 1354 unique DEGs. Hierarchical clustering of the DAR accessibility signal clearly separated the different myeloma MIE subgroups from each other (Fig. 2a). While this showed a group of regions where changes correlated with MIE, it also highlighted accessibility changes that are shared between different myeloma subgroups. The MAF subgroup for instance, demonstrated the highest number of distal DAR that are >2-fold different from ND, some of which were unique to MAF while others were also >2-fold more accessible in other genetic subgroups. To validate this further, we clustered myeloma PC (*n* = 26) from a previous study^11^ using these same 2581 regions, and confirmed that these samples mostly clustered with samples carrying the same MIE from this study (Supplementary Fig. 3a).

Of note, 977 of the 2581 (38%) DAR were proximal to more than one DEG, while 962 of 1354 DEG (71%) were within 1 Mb of more than one DAR (Supplementary Data 4), and often in more than one genetic subgroup. These findings suggest that diverse MIE functionally converges to aberrantly regulate the same regions of chromatin, a process consistent with chromatin accessibility-based convergence evolution.

At least 14 of the DEG linked to distal DAR have been implicated in myeloma pathogenesis, including *HGF*^16^, *DKK1*^17^ and *UCHL1*^18^ (Fig. 2b, Supplementary Fig. 3b and c and Supplementary Data 4). In two myeloma cell lines studied, these same regions are marked by H3K27ac, a histone hallmark of active enhancers, and therefore in terms of chromatin status they can be considered as bona fide enhancers.

The 1354 unique DEGs linked to 2581 unique DAR showed overall enrichment for previously defined myeloma transcriptional signatures (Supplementary Fig. 4a), and amongst other pathways, they were also notably enriched for the oncogenic RAS pathway, activated in 40% of MM patients^3,19^ (Fig. 2c and Supplementary Data 5a). There was also enrichment of the Hedgehog pathway, previously implicated in the regulation of *CCND1* and *CCND2*^20^ (Fig. 2c) an over-representation of genes marked by H3K27me3 and Polycomb repressive complex components in several cell types (Supplementary Fig. 4b, Supplementary Data 5b) and enrichment for genes selectively expressed in neuronal cell types, particularly in the HD and MMSET subgroups (Supplementary Fig. 4b and c, Supplementary Data 5c).

Finally, enrichment of previously validated MIE geneset classifiers was highest in their corresponding genetic subgroups (Fig. 2d).

Together, these findings suggest that DAR with regulatory potential are to a large extend shaped by MIE, although secondary genetic events are also likely to contribute.

### Developmentally ‘re-commissioned’ and de novo formed enhancers in MM

Next, we sought to gain insights into the developmental origins of the MM over-accessible candidate enhancers linked to DEG. For this purpose, we tracked the candidate enhancer chromatin status, as defined by combinatorial enrichment of histone marks (ChromHMM states^21^), across different mature B lineage cells^22^ (Fig. 2e and Supplementary Data 6).

Considering that an active enhancer requires combined enrichment for H3K27ac with H3K4me1^23^, we identified 254 (out of 832) DAR with predicted regulatory activity over 201 DEG within the same TAD that are only present in myeloma and not normal PC (Fig. 2e and Supplementary Data 6), i.e., they are de novo formed (e.g., the enhancers predicted to regulate *HGF* and *UCHL1* shown above). TF motif enrichment analysis of these 254 DAR identified IRF and MEF families of TF amongst others as possible leading transcriptional regulators of their activity (Fig. 2f). In addition, a smaller number of DAR are ‘re-commissioned’ in myeloma PC, i.e., active in one or more B lineage cells but inactive in ND PC (Supplementary Data 6).

Therefore, biochemical annotation of the distal over-accessible chromatin profile identifies enhancers that are myeloma PC unique or developmentally inherited.

### TF ‘rewiring’ reveals myeloma dependencies with prognostic impact

**Fig. 3:**
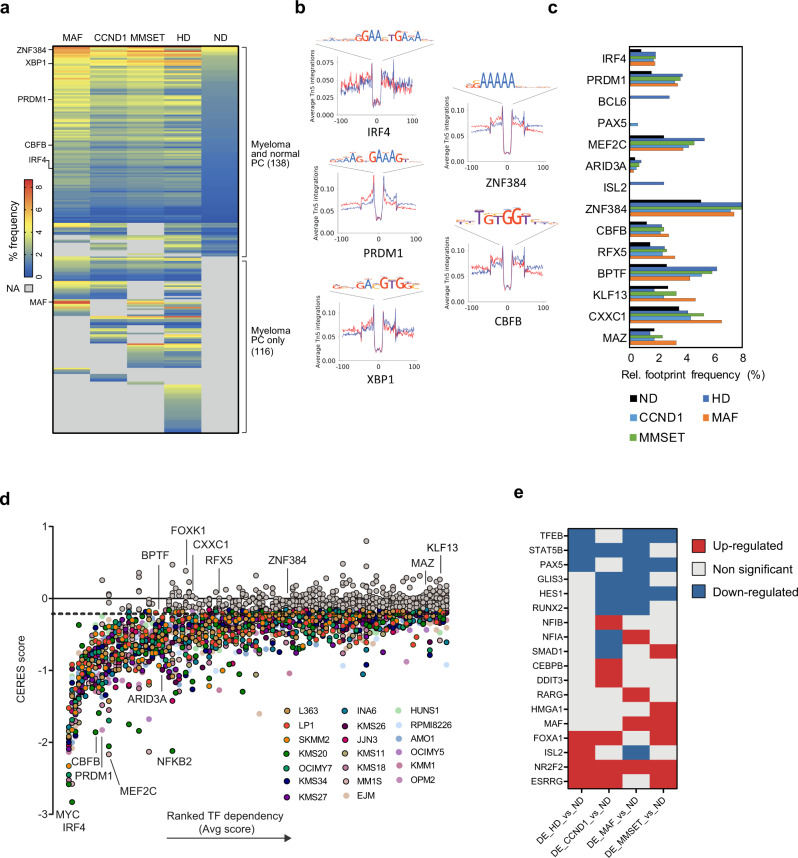
‘Rewiring’ of transcription factors underpins aberrant regulatory gene networks in MM. **a** Heatmap representation of the relative frequency of TF footprints across different MM genetic subgroups. TF are clustered based on their presence in both normal donor and myeloma PC (top) or in myeloma PC only (bottom). Grey values indicate lack of TF expression and/or predicted binding. **b** Footprints of indicated TFs identified in HD myeloma PC as determined by ATAC-seq. **c** Difference in relative footprint frequency of active TFs between different myeloma subgroups and normal donor PC. **d** TF dependency analysis using high-throughput CRISPR screens data from DepMap database (20 myeloma cell lines representing CCND1, MAF and MMSET genetic subgroups; colour-coded). Of the 247 TF shown, 137 are predicted to generate dependency, the top 100 of which are shown here. Examples of established and novel (bold) dependencies are indicated. In this analysis, dependency is defined as CERES score < −0.2 (horizontal dotted line) in at least 4/20 myeloma cell lines. **e)** TF that are differentially expressed between ND PC and myeloma genetic subgroup PC.

Next, we employed ATAC-seq footprinting^24^ to identify the predicted association of DNA binding factors with chromatin across myeloma and ND PC. In total, 138 of 254 expressed TF (Fig. 3a and Supplementary Data 7a–c) displayed higher or similar predicted binding frequency in at least one myeloma subgroup compared to ND PC, and included TF such as XBP1, IRF4 and PRDM1 known to regulate myeloma transcriptomes, but also TF such as CBFB and ZNF384 which have not been previously linked to myeloma biology (Fig. 3a, b and c). Another 116 TFs were predicted to bind to chromatin in at least one MM subgroup but not in ND PC, including established, subgroup-specific oncogenic drivers (e.g., MAF). Almost one third of these 116 TF were predicted to be exclusively active in individual subgroups, with ISL2, a neural TF^25^ not previously linked to MM, showing activity solely in the HD subgroup **(**Fig. 3a–c, and Supplementary Data 7b).

CRISPR/Cas9 screens involving depletion of 247/254 TF as retrieved from the DepMap database, suggested myeloma cell dependency on 55% (137/247) of TF in at least 4/20 myeloma cell lines at a CERES score of <−0.2 (Fig. 3d and Supplementary Data 7d), and confirmed MM cell dependency on the TF *CBFB* and *ZNF384* identified by ATAC-seq footprinting (Fig. 3c and d). Interestingly only 13% (18/137) of these TF are differentially expressed in one or more myeloma subgroups compared to ND PC (Fig. 3e). This is consistent with a pattern of TF activity ‘re-wiring’ in myeloma PC that does not necessarily require transcriptional deregulation of the TF themselves.

**Fig. 4:**
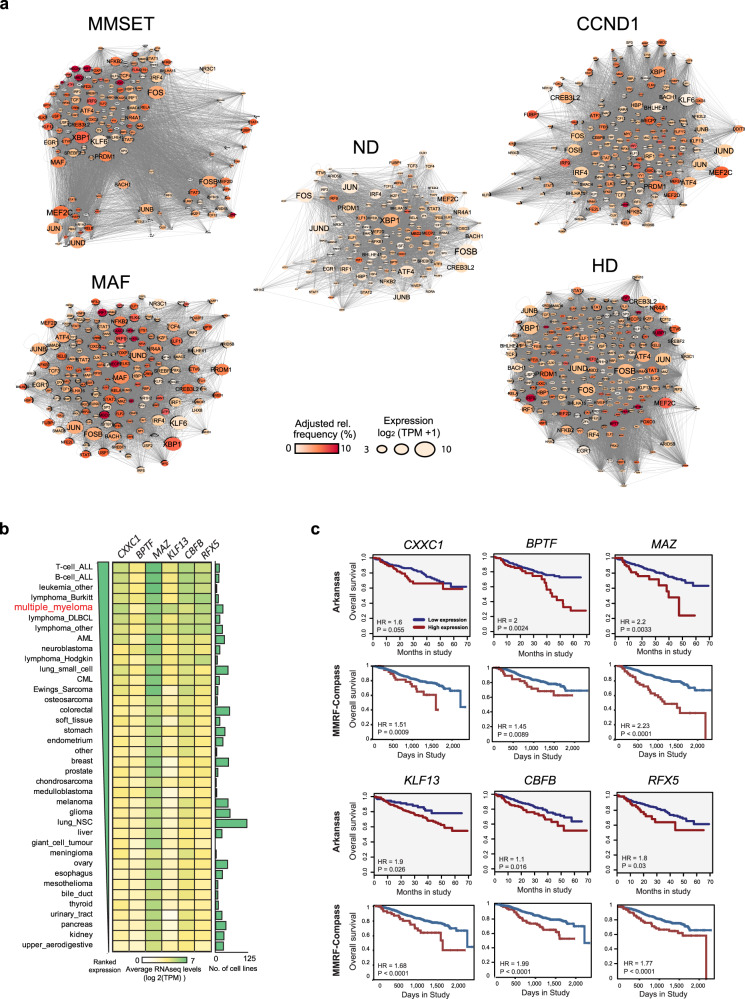
TF regulatory gene networks provide biological and clinical insights into MM disease. **a** TF regulatory gene networks per myeloma subgroup and normal donor PC, as inferred from footprinting analysis. TF are weighed by relative TF binding frequency (colour) and expression (node size). **b** Heatmap of ranked expression of *CXXC1*, *BPTF*, *MAZ*, *KLF13, CBFB* and *RFX5* across >1000 human cancer cell lines (CCLE dataset). Multiple myeloma cell lines are highlighted in red. Bar plot depicts the number of cell lines per cancer group. **c** MM patient stratification based on *CXXC1*, *BPTF*, *MAZ*, *KLF13, CBFB* and *RFX5* expression (red, high; blue, low) and analysis of overall survival using the Multiple Myeloma Arkansas (*n* = 414) and the MMRF Compass Dataset (*n* = 745). HR hazard ratio.

To define this ‘re-wiring’ of TF further, for each MM subgroup and ND PC we built TF regulatory gene networks based on the weighted frequency of binding and level of expression (Fig. 4a). In general, compared to ND PC, we observed a higher number of active TF in all myeloma subgroups. These formed higher density regulatory connections with other TF and displayed auto-regulatory loops (Supplementary Fig. 5a and Supplementary Data 7e), commensurate with increased binding frequency for >90% of TF in each myeloma subgroup (Supplementary Fig. 5b). For example, the established myeloma cell dependencies and PC lineage-defining TF IRF4, XBP1 and PRDM1, are also predicted to be connected to a higher number of other TF in HD, MMSET and CCND1 subgroups than in ND PC, while TF of the MEF family that have not been linked to myeloma biology before and are predicted to regulate de novo formed myeloma enhancers (Fig. 2f**)** display higher connectivity and betweenness (centrality). A high level of myeloma cell line dependency to MEF2C in the DepMap CRISPR/Cas9 screen (Fig. 3d), highlights an important role of this TF in the biology of MM, least because of its inferred role in activating transcription of TF such as IRF4, XBP1 and PRDM1 (Supplementary Data 7f).

Focusing on the MAF-translocated subgroup, we performed ChIP-seq to obtain the cistrome of oncogenic MAF in the MAF-translocated myeloma cell line MM.1S (Supplementary Fig. 5c). MAF binding was predominantly enriched at TSS/promoters and intergenic areas (Supplementary Fig. 5d). Motif analysis performed on the MAF binding sites identified significant enrichment of the MAF motif (Supplementary Fig. 5e) and motifs of IRF1-4, NRF2/NFE2L2, ATF3, BACH1 TFs (Supplementary Fig. 5f), which were also predicted to be active within the MAF subgroup gene regulatory network (Supplementary Data 9b).

Six TFs (CXXC1, BPTF, MAZ, KLF13, CBFB and RFX5) were identified as showing higher connectivity in all myeloma subgroups compared to ND PC (Supplementary Data 7e), thus exemplifying the process of existing TF ‘re-wiring’ in MM. These TF, none of which have been previously linked to MM, share another three notable features: (a) they demonstrate dependency on DepMap upon CRISPR/Cas9 and siRNA screens (Fig. 3d and Supplementary Fig. 5g), (b) display highest expression in multiple myeloma cell lines compared to >1000 other cancer cell lines; and (c) their higher expression has a significant adverse impact on survival in two independent myeloma patient cohort datasets (Fig. 4b and c). In addition, myeloma cell dependency on *CXXC1* was further validated by independent shRNA knockdown experiments (Supplementary Fig. 5h). Together, this multi-layered approach reveals TF dependencies and prognostic variables in MM.

### Identification and characterisation of the *CCND2* super-enhancer

Furthermore, we sought to identify and characterise the regulatory mechanisms of *CCND2* overexpression in MM.

**Fig. 5:**
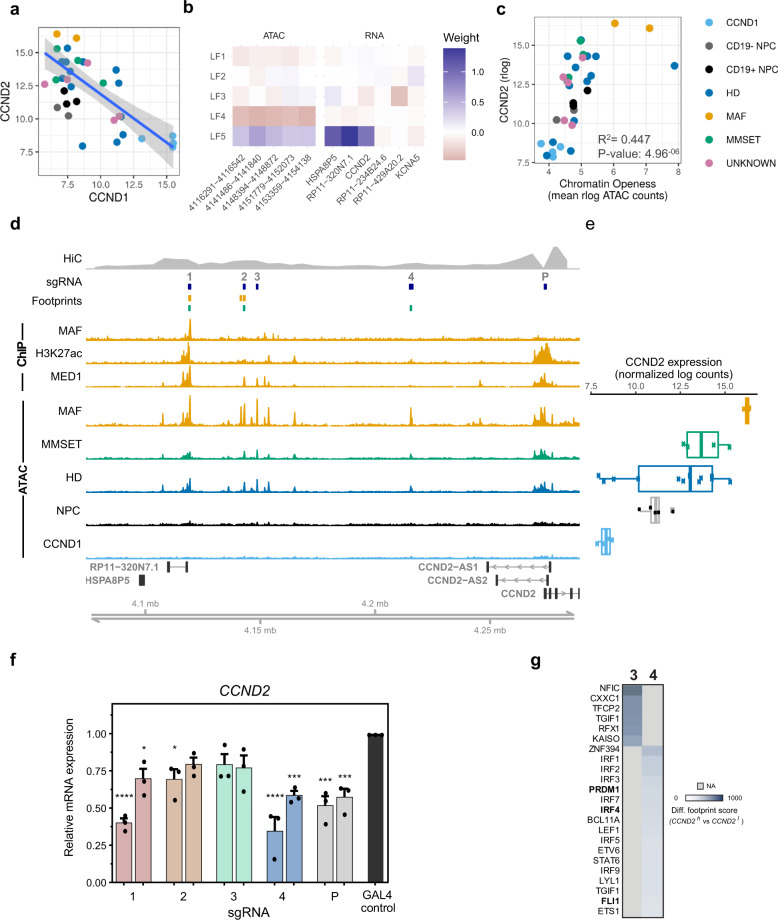
A super-enhancer regulates transcription of *CCND2* in myeloma. **a** Inverse correlation between expression of *CCND1* and *CCND2* across all MM and normal PC samples. The best linear regression fit is indicated as a blue line with standard error in grey. **b** Loadings from MOFA analysis for 5 regions and 6 genes within 1 Mb of *CCND2* that were selected for MOFA analysis for latent factors 1–5. Both regions and genes ordered in a 5’->3’ direction. **c** Average chromatin accessibility of regions upstream of *CCND2* and *CCND2* gene expression across all samples in this study. **d** Genomic tracks visualization of the *CCND2* region. From top to bottom: Hi-C signal for interactions with *CCND2* promoter in GM12878 B cells; target location of sgRNAs designed for the CRISPRi experiment; Predicted footprints for MAF TF in MAF-translocated (orange) and MMSET-translocated (green) samples; ChIP-seq signal against MAF, H3K27ac and MED1 in MM.1S cells; normalised ATAC-signal in each subtype, ordered by mean *CCND2* expression. **e** *CCND2* expression as assessed by RNA-seq in samples across different MM subgroups and normal donor PC. Subgroups as indicted in adjacent tracks in (**d**). Boxplots display all values as points, whisker’s box (min to max) and mean expression per subgroup. **f** *CCND2* expression as assessed by qPCR 4 days after CRISPRi of *CCND2* super-enhancer and promoter regions. Two sgRNAs were employed to target the promoter (P) and each of four major peaks (1–4) of the *CCND2* super-enhancer, as shown in (**d**), and compared with a non-targeting control (Gal4). Error bars represent mean + SEM for three independent experiments. Statistical analysis: one-way ANOVA with Dunnett’s post-hoc multiple comparisons correction. *p < 0.05, ***p < 0.001, ****p < 0.0001. **g** Heatmap illustration of TF motifs significantly enriched in *CCDN2*^high^ versus *CCND2*^low^ HD samples, as identified by differential footprinting analysis. Differential footprints were identified in peaks 3 and 4 (see Fig. 5d).

The LF5 of MOFA analysis completely separated *MAF*-translocated from *CCND1*-translocated samples (Fig. 1f and g), placing extreme opposite weights on the expression of *CCND2* and *CCND1*, respectively (Fig. 5a). LF5 also places high importance on a set of open-chromatin regions upstream of *CCND2*, linking them to enhanced expression of *CCND2* and transcripts upstream, but not downstream, of *CCND2* (Fig. 5b). Accessibility of this region correlates with CCND2 activity irrespective of MIE (Fig. 5c). Further, super-enhancer calling using H3K27ac and MED1 chromatin marks in *MAF*-translocated MM.1S myeloma cells identified the region of interest as a bona fide super-enhancer by both markers (Fig. 5d and Supplementary Fig. 6a).

Consistent with it being a ‘re-commissioned’ enhancer of *CCND2*, the region of interest is Polycomb repressed in GCB and PC but active in naïve and memory B cells (Supplementary Fig. 6b); accordingly, *CCND2* is expressed in naïve and memory B cells but not GCB cells or PC (Supplementary Fig. 6c).

Using Hi-C genome data from GM12878 B cells^13^, we identified exclusive, long-range interactions of the *CCND2* promoter with the upstream accessible clusters of the putative enhancer (Fig. 5d and Supplementary Fig. 6d). In a complementary approach, we employed KRAB-dCAS9 CRISPRi in MAF-translocated myeloma cells to repress the activity of four prominent constituent peaks 1–4 (Fig. 5d) which engage in high-frequency interactions with the *CCND2* promoter. As expected, targeting a promoter accessibility peak resulted in a significant decrease in *CCND2* expression, while in the *CCND2* enhancer, the most pronounced effect, similar to that of the promoter peak, was conferred by targeting the proximal peak 4 and distal peak 1 accessibility regions **(**Fig. 5f**)**. Notably, accessible peaks 1 and 4, but not others in between, are Polycomb-repressed in GCB and PC but active in naïve and memory B cells (Supplementary Fig. 6b). Thus, the relative importance of peaks 1 and 4 is also validated from a developmental perspective.

Having dissected the *in cis* regulatory mechanisms of *CCND2* expression, we proceeded with the characterisation of *trans* factors involved in this process.

Previous work showed that MAF binds to *CCND2* promoter in vitro^26^, providing some insight into how *CCND2* is regulated in the MAF genetic subgroup. Importantly, our ChIP-seq analysis in MM.1S myeloma cells shows that MAF binds to the enhancer of *CCND2* in vivo (Fig. 5d), thus consolidating its role as a critical regulator of *CCND2* over-expression in *MAF*-translocated MM cells. This finding provides also insights into *CCND2* regulation in the MMSET genetic subgroup, in which, both *CCND2* and *MAF* are expressed at lower levels^26^ (Fig. 5e and Supplementary Fig. 6e). Since chromatin accessibility signal is also lower in the MMSET than in the MAF subgroup (Fig. 5d and Supplementary Fig. 6e), together, these findings are consistent with the notion that the transcriptional activity of *CCND2* enhancer in the MAF and MMSET subgroups is MAF dosage-dependent.

To identify other TF potentially regulating *CCND2* enhancer activity in *CCND2*-expressing HD MM (which lack expression of MAF), we performed differential footprinting analysis in CCND2^high^ vs CCND2^low^ HD myeloma PC (Fig. 5g and Supplementary Fig. 6f and g). In addition to TF known to be implicated in MM (IRF4, PRDM1, FLI1)^11,27^, we also identified a potential regulatory role for TF previously not linked to MM (e.g., CXXC1, ZNF394, and IRF3).

Finally, we explored the activity of the *CCND2* super-enhancer in B cell chronic lymphocytic leukemia (CLL), the most common blood cancer (Supplementary Fig. 7a and b). High CCND2 expression has been previously documented in CLL B cells residing in proliferation centers^28^, structures in secondary lymphoid organs where malignant B cells receive survival and proliferative signals^28^. CLL malignant B cells express significantly higher *CCND2* than *CCND1* levels (Supplementary Fig. 7b) and as in *CCND2*^high^ myeloma PC, we found that the same *CCND2* super-enhancer is active in CLL B cells (Supplementary Fig. 7a). This observation extends the importance of the *CCND2* enhancer in a wider range of B cell lineage malignancies.

## Discussion

Our analysis of complementary datatypes sheds insights into how changes in the regulatory genome, and in particular of the MM-specific enhanceome, shape distinct myelomagenic transcriptomes and downstream biological pathways.

Increased overall chromatin accessibility in all myeloma genetic subgroups with reference to normal BM PC, the myeloma PC normal counterparts, is our first fundamental observation. Unlike previous studies, here we addressed chromatin and gene expression-derived heterogeneity in MM. Surprisingly, we found that chromatin accessibility explained more of the variance than gene expression. The main axes of variation correlated surprisingly well with ND/MM and MIE categorizations, and defining subgroups as clusters, the combined model provided better separation than either data type alone. This was possible for two of the three IgH-translocated subgroups, and less so for HD MM, likely because the latter represents an inherently biologically heterogenous group. While the small number of samples in each subgroup argues for caution in interpreting this, we were able to validate these axes with independent data from cell lines not used in training the model. While using peaks found in the primary samples to assess cell lines is likely to overemphasise the similarity of cell lines and primary samples overall, it should not make specific cell line subtypes appear more like their primary counterparts. Larger future datasets will undoubtedly be helpful for increasing further our confidence in difference between the programs associated with different MIE. We found that marker genes and genesets that have been previously shown to be regulated by MIE, are also correlated with distal DAR of myeloma PC chromatin. This suggests that such chromatin changes are, to a large extent, directly or indirectly, dependent on MIE. Nevertheless, some of the identified putative enhancers are predicted to regulate genes downstream of the oncogenic RAS pathway which is activated by secondary gain-of-function *N-* or *K-RAS* somatic mutations in 40–50% of MM cases^3,19^, thus highlighting the ability of our approach to also reveal chromatin traces of secondary driver genetic events. Future studies, specifically designed to address the impact of pre-defined, high-frequency secondary genetic events (e.g., *RAS* mutations and *MYC* structural variants), will reveal the extent to which such secondary events impact chromatin and its regulatory activity. Another notable feature, mostly restricted to HD and MMSET MM, is the proximity of putative enhancers to genes involved in neurogenesis with ectopic expression of the TF *ISL2* in HD MM being a prime example of this. While the functional significance of this observation requires further investigation, we note that recent work demonstrated ectopic activation of neural genes in different cancers with such genes engaging in functional cross-talk with the central and peripheral nervous system^29,30^.

By taking advantage of a comprehensive set of chromatin marks in the whole spectrum of B lineage cells, we validated the presence of H3K27ac in 832 over-accessible, candidate enhancer regions in myeloma PC. The candidate enhancers of *HGF* and *UCHL1* exemplify de novo formed enhancers, i.e., not present at any stage of late B lineage development, while the *CCND2* enhancer provides an example of ‘re-commissioned’ super-enhancer, i.e., active in myeloma but not in normal PC. In the case of *CCND2*, ‘recommissioning’ entails activation in myeloma PC of Polycomb-imposed ‘poised’ transcriptional states in GCB cells and normal PC.

As well as discovery of critical *cis* regulatory elements, ATAC-seq also affords the opportunity for inferring TF binding to chromatin through footprinting and motif analysis. A notable finding is that the majority of *trans* factors that are predicted to display increased binding frequency to chromatin are already active in ND PC and their expression is not deregulated in MM. Such TF engage at higher frequency interactions with other TF than in ND PC and are more likely to self-regulate, properties that have been associated with enhanced regulatory potential^31^. These properties, as revealed through construction of TF regulatory gene networks, led to insights with functional and prognostic implications. In the case of MEF2C, the set of its predicted target TF, includes archetypical myeloma PC dependencies, i.e., IRF4, PRDM1, thus in part explaining the high degree of myeloma cell dependency on MEF2C. Similarly, myeloma cells appear to be addicted to CBFB and ZNF384 which have not been linked to myeloma PC biology thus far. In addition, the case of CXXC1, BPTF, MAZ, KLF13, CBFB and RFX5, six TF with unknown function in MM, also highlights the strength of our functional epigenomics approach. None of these TF is differentially expressed in MM, yet they display increased predicted regulatory potential across all myeloma subgroups, likely reflecting their high expression levels in myeloma cells compared to other cancers. Moreover, all six TF demonstrated prominent myeloma cell dependency and were found to strongly predict prognosis. For CXXC1, this is in accordance with its known function in the regulation of H3K4me3 as part of the COMPASS activating complex and through its binding to CpG islands and interaction with the histone methyl-transferases SETD1A/B^32,33^. Although little is known about the role of CXXC1 in cancer, it has been reported that its overexpression portends adverse prognosis in all stages of gastric cancer^34^. Therefore, the inferred TF networks provide a firm basis for future research which will further validate and define the role of TF identified herein in myeloma biology.

Extensive genetic heterogeneity and diversification in MM poses significant therapeutic challenges. One of the striking features of myeloma biology is the early observation that a dichotomous over-expression of the cell cycle regulators *CCND1* and *CCND2* overarches genetic diversification^4^. While in the majority of MM cases overexpression of *CCND1* can be explained by somatic structural variants i.e., juxtaposition to IGH enhancer or chr11q25 gain^31^, such aberrancies do not account for overexpression of *CCND2*. Our identification and functional validation by complementary approaches of the distal *cis* and *trans* regulators of *CCND2* expression addresses this gap in the biology of MM and allowed further insights into how the activity of this enhancer is regulated in different myeloma genetic subgroups. While expression of *MAF* is highest in the MAF subgroup as a result of its juxtaposition to the powerful *IgH* enhancer, in MMSET MM expression of *MAF* is lower and previously shown to be regulated by the TF FOS in response to activated MAPK pathway^35^. It is likely that these differences in MAF dosage account for the strongest chromatin accessibility signal at the MAF-bound *CCND2* enhancer and a higher level of *CCND2* expression in MAF-translocated MM. Interestingly, the same enhancer with the same developmental chromatin features regulates expression of *CCND2* in CLL cells, a finding that tallies with the notion that CLL originates either from a naïve or memory rather GCB cell^36^.

Overall, our dissection of the MM regulatory genome offers insights and a resource for further biological exploration. Discovery and subsequent functional dissection of the critical *CCND2* enhancer is a prime example of the power of our multi-layered integrative computational approach and affords opportunity for enhancer-based therapeutic approaches.

## Methods

### Ethics statement

Bone marrow aspirates were obtained in accordance with the criteria of the Declaration of Helsinki and following written informed consent and national research ethics committee (REC reference: 11/H0308/9) and Imperial College London Joint Research Office approval.

### Patient and normal donor samples

Patient BM aspirates were subjected to red cell lysis. Multiple myeloma PC were purified by two rounds of CD138 immunomagnetic selection (Miltenyi Biotech) following the manufacturer’s instructions. Pre- and post-selection purity was assessed by FACS analysis (BD LSR-Fortessa) using CD138, CD45, CD19, CD56 and CD38 markers (Supplementary Table 1 and Supplementary Fig. 1a), at 1:100 antibody dilution Purified cells were immediately processed for ATAC-seq and RNA-seq.

Normal donor BM mononuclear cells from BM aspirates (BM-MNCs) were isolated by Ficoll (Histopaque, Sigma). The BM-MNCs were pre-cleared of T cells and Monocytes by consecutive immunomagnetic negative selection (CD3 and CD14-EasySep StemCell Technologies) following the manufacturer’s instructions. The samples were stained (1:100 antibody dilution) and sorted for CD138+, CD319+, CD27+, CD45+ and CD38+ (positive), for CD2−, CD3−, CD14−, CD16−, GPA− and 7AAD- (negative) and CD19+/− (positive or negative, Supplementary Table 1 and Supplementary Fig. 1a) (FACSAriaII, BD Biosciences). Sorted cells were immediately processed for ATAC-seq and RNA-seq.

All FACs antibodies have been extensively validated by the manufacturers.

### Fluorescence in situ hybridisation (FISH)

FISH was undertaken using a panel of 4–7 probe sets targeting regions of common cytogenetic abnormalities in multiple myeloma (Kreatech Diagnostics, Amsterdam, The Netherlands). Interphase cells were dropped onto a glass slide and dried briefly before fixation in situ. Hybridisation was performed according to the manufacturer’s protocols. The panel consists of two probes (13q14 and 13qter) to detect deletion and monosomy of chromosome 13, a locus specific probe to detect deletion of TP53 (17p13), two probes on chromosome 9 and 15 to detect HD and a dual colour, break-apart probe to detect rearrangements of IGH (14q32). Rearrangements of IGH were further investigated with IGH/CCND1, IGH/MAF and IGH/FGFR3 dual colour, dual fusion probes. The upper threshold for normal results is according to probe type (dual colour break apart 5%; quantitative 5%; dual colour dual fusion 2%). In all cases, a minimum of 50 interphase cells were scored by two independent analysts.

### Cell lines

The human multiple myeloma cell line (MMCLs) MM.1S, NCI-H929 (ATCC, Manassas, VA, USA), U266, KMS12BM and OPM2 (DSMZ, Germany) were cultured in RPMI1640 media (Sigma, UK) and 20% FBS (Life Technologies). JJN3 cells (DSMZ, Germany) were cultured in 40/40% DMEM/IMEM medium (Sigma, UK), and 20% FBS. HEK 293T (ATCC, Manassas, VA, USA) cells were cultured in DMEM (Sigma, UK), 10% FBS (Gibco). All cell lines were maintained at 37 °C and 5% CO2 and the growing media was supplemented with 1% penicillin/streptomycin (Sigma, UK) and 1% L-glutamine (Sigma, UK). Testing for mycoplasma presence was performed every 4 weeks.

### ATAC-seq

ATAC-seq was performed on purified normal donor or myeloma patient samples^37^. Briefly, 50,000 purified PC, myeloma PC or cell lines, were washed with cold PBS (Sigma, UK) at 500 × *g* at 4 °C for 5 min. The cells were resuspended in 50 μL of cold Lysis Buffer (10 mM Tris-HCl, pH 7.4, 10 mM NaCl, 3 mM MgCl_2_, 0.1% IGEPAL CA-630) and washed at 500 g at 4 °C for 10 min. The nuclei were subjected to transposase reaction for 30 min at 37 °C; termination of the reaction and DNA purification was performed using a MiniElute Kit (Qiagen) and eluted twice with 10 μL. The purified DNA was amplified as described before with NEBNext High-Fidelity 2x PCR Master Mix (New England Biolabs). The PCR amplified product was cleaned twice with (0.9X) AMPure beads (Beckman). The quality of the libraries was assessed with the Bioanalyzer High Sensitivity DNA kit (Agilent). The libraries were quantified using the NEBNext Library Quant Kit for Illumina (New England Biolabs) on a StepOne Plus Real-Time PCR (Applied Biosystems). The libraries were sequenced at the Genomics Facility at ICL using the Illumina HiSeq 4000 platform to obtain paired-end 75 bp reads.

### RNA-extraction and cDNA synthesis qPCR

Purified PC, myeloma PC or cell lines (100,000), were washed with cold PBS (Sigma, UK) at 500 × *g* at 4 °C for 5 min. Total RNA was isolated using the Nucleospin RNA kit (Macherey-Nagel) and quantified by Nanodrop Lite (Thermoscientific). cDNA was synthesized with RevertAid cDNA synthesis kit (Thermoscientific). qRT-PCR was performed with Taqman probes (Applied Biosystems) using StepOne Plus Real-Time PCR (Applied Biosystems). Gene expression was normalized to the expression of *GAPDH* (Supplementary Table 2). Taqman probes: *GAPDH* (Hs03929097_g1), *CCND2* (HS00153380_m1), *IRF4* (Hs01056535_m1), *CXXC1* (Hs00969402_g1).

### RNA-seq

Total RNA was isolated using the Nucleospin RNA kit (Macherey-Nagel) and quantified using the Qubit RNA Assay kit (Life Technologies) and RNA quality was assessed on the Bioanalyser using the RNA pico kit (Agilent). Total RNA libraries were prepared by removing the ribosomal RNA with NEBNext rRNA depletion kit (New England Biolabs) and NEBNext Ultra II Directional RNA Library Prep kit for Illumina (New England Biolabs), following the manufacturer’s instructions. Library quantity was determined using the Qubit High Sensitivity DNA kit (Life Technologies) and library size was determined using the Bioanalyser High Sensitivity DNA kit (Agilent). Libraries were diluted to 2 nM and sequenced using the Illumina HiSeq 4000 platform the Genomics Facility at ICL to obtain paired-end 75 bp reads.

### ChIP-seq

For ChIP-seq, MM.1S cells were cultured using RPMI-1640 medium^38^. 3–5 × 10^7^cells were pelleted by centrifugation at 300 × *g*, washed with PBS and crosslinked with 1% formaldehyde (Sigma, UK) for 15 min. Crosslinking was stopped by adding glycine to a final 125 mM. Cells were washed 3× with cold PBS. The cells were lysed (50 mM Tris-HCl pH:8.1, 1% SDS, 10 mM EDTA (pH:8), and 1× protease inhibitors (Sigma, UK) 20 min at 4 °C. Nuclei were sonicated at 4 °C in a Bioruptor UCD-200 (Diagenode).

Post-sonication fragments of average 500–300 bp length were confirmed on a 1.5% agarose gel. The chromatin was diluted at least 10 times in ChIP dilution buffer (0.01%SDS, 1.1% Triton X-100, 1.2 mM EDTA, 16.7 mM Tris-HCL pH 8, 167 mM NaCl) containing 1× protein inhibitors (Sigma, UK). The diluted chromatin was pre-cleared by incubation with BSA-blocked magnetic beads (Dynabeads Protein A + G form Invitrogen) for 1 h at 4 °C on a rotating wheel. Pre-cleared chromatin was incubated with 2–5 μg of antibody, at an approximate dilution of 5:1000 (Supplementary Table 1), overnight at 4 °C on a rotating wheel. Protein A + G magnetic beads were added and incubated for 2–4 h at 4 °C on a rotating wheel. The immunoprecipitates were washed for 5 min at 4 °C in a rotating wheel with 2X low salt buffer (0.1% SDS, 1% Triton X-100, 2 mM EDTA, 20 mM Tris-HCl pH 8, 150 mM NaCl), 2X high salt buffer (0.1% SDS, 1% Triton X-100, 2 mM EDTA, 20 mM Tris-HCl pH 8, 500 mM NaCl), 2X LiCl buffer (0,25 M LiCl, 1 % IGEPAL, 1% sodium deoxicolate, 1 mM EDTA, 10 mM Tris-HCl pH 8) and two times with TE.

Immunocomplexes were eluted by adding 150 μl elution buffer (50 mM Tris-HCl pH 8, 50 mM NaCl, 1 mM EDTA and freshly added 1% SDS and 20 mg/ml of RNaseA) at 65 °C for 4 h to overnight and a second time for 30 min. Both elutions were pooled and treated with proteinase K (Thermoscientific).

The DNA was purified using AMPure beads 1:1.8X ratio. ChIP and input DNA libraries were prepared using the NEBNext ULTRA II ChIP-seq Library kit for Illumina (New England Biolabs) following the manufacturer’s protocols. The quantity was determined using the Qubit High Sensitivity DNA kit (Life Technologies) and library size was determined using the Bioanalyser High Sensitivity DNA kit (Agilent). Libraries were sequenced using the Illumina HiSeq 2500 platform to obtain single-end 50 bp reads. The H3K27Ac ChIP antibody has been extensively validated by the manufacturers. In addition, all ChIP antibodies are validated by ChIP qPCR, against IgG control. In the case of the MAF antibody, cell lines not expressing the protein were used as additional negative controls.

### Cloning of dCas9-KRAB CRISPRi vectors

Two single guide RNAs (sgRNAs) targeting each peak were designed using the online tool http://crispr.org (Supplementary Table 2). Two sgRNAs were designed for the *CCND2* promoter as a positive control and the non-targeting sgRNA for *Gal4* of the yeast *S.cerevisiae* as a negative control.

The vector used was the inducible Lenti-CRISPR-dCas9-KRABv2. The original LentiCRISPRv2 vector (Addgene 52961, USA) was modified and kindly supplied by Dr Niklas Feldhahn (Imperial College London). The plasmid was digested with Esp31 (New England Biolabs), removing a 2 kb stuffer.

The sgRNA oligos were phosphorylated and annealed using the T4 Ligation Buffer (New England Biolabs) and T4 PNK (New England Biolabs). The sgRNAs were then ligated with the digested plasmid using the T4 DNA ligase and buffer (New England Biolabs). Reactions were carried out following the Zhang Lab General Cloning Protocol (Addgene).

Sanger sequencing was used to confirm the exact sequence of the cloned sgRNA (GeneWiz Ltd UK).

### Cloning of pLKO.1-GFP shRNA vectors

The pLKO.1-GFP was obtained by replacing the selection marker puromycin with eGFP cDNA in the pLKO.1-PURO lentiviral vector (Addgene plasmid #27994). Two independent shRNA oligoes (Supplementary Table 2) were annealed and cloned into AgeI/EcoRI digested pLKO.1-GFP vector^38^.

Successful cloning of shRNA sequences was confirmed by Sanger sequencing (GeneWiz Ltd UK).

### Lentivirus production and cell transduction

The dCas9-KRAB and pLKO.1-GFP constructs were transfected in 293T cells with the 2nd generation lentiviral packaging and envelope plasmids psPAX2 and pMD2.g (Addgene, USA), using CaCl_2_ 2.5 M (Sigma, UK) and 2x HEPES-Buffered Saline, pH = 7 (Sigma, UK). After 10 h, cells were incubated with glycerol (15% v/v) (Honeywell, USA) for 3 min. Cells were washed with PBS (Sigma, UK) and then incubated with fresh DMEM medium (Sigma, UK) for 36 h. The virus was harvested, filtered (0.45 μm) and concentrated by ultracentrifugation (23,000 rpm for 1 h and 40 min, at 4 °C) 48 h and 72 h post transfection using the Thermo Sorvall Ultracentrifuge, MTX (Applied Biosciences). Transduction of JJN3 MMCL for dCas9-KRAB and MM.1 S and H929 cells for pLKO.1-GFP was performed in the presence of 8 μg/ml polybrene (Sigma, UK). For CRISPRi experiments, JJN3 cells were selected 3 days post-transduction with puromycin (5 μg/ml), and 48 h later viable cells were sorted on MA900 Multi-Application Cell Sorter (Sony Biotechnology) and cultured in fresh DMEM/IMEM medium (Sigma, UK). Cells were grown for 10 days, before induction with doxycycline (Sigma, UK) topped up daily to a final concentration of 1 μg/ml. On day 4, GFP+ cells were FACS sorted for RNA extraction. For shRNA knockdown experiments, transduced cells (GFP+) were assessed 3 days after transduction and after every 2-3 days by flow cytometry with a BD LSR FORTESSA analyser. To confirm successful knockdown, GFP+ cells were sorted on MA900 Multi-Application Cell Sorter (Sony Biotechnology) 4 days after transduction. Total RNA was isolated and retro-transcribed as described above.

### Computational analysis

Unless otherwise stated, the human genome version hg38 with alternative contigs removed was used for all analyses, and annotations were taken from Ensembl version 85. Sample ND3- from Supplementary Data 1 did not pass the quality control and was not included in the analysis.

### ChIP-seq analysis

ChIP-seq data alignment was performed with Bowtie2 using the default settings and duplicate reads in bam files were removed with Picard *MarkDuplicates*. Peak calling was performed using MACS2 with the following settings: for TFs, narrow peaks were detected using the following settings: *-B -q 0.01–verbose 4–SPMR–call-summits*; for histone-marks, broad peaks were obtained using the parameters: *-B–broad–broad-cutoff 0.01–verbose 4–SPMR*. Genome browser tracks were created using the *deeptools* commands set, with the signal values normalized to fold change against input. Tools from Homer package (v4.9) were used for motif analysis, super-enhancer calling and annotation of genomic regions against the hg38 human genome, following the default mode.

### RNA-seq analysis

Read cleaning and filtering—Paired-end RNA-seq reads were adapter trimmed by the DNA sequencing facility at Centre for Haematology, Division of Experimental Medicine Faculty of Medicine, Imperial College London were quality controlled with FastQC version 0.11.3.

RNA-seq quantification—Expression estimates for each sample were obtained using Salmon version 0.11.4^39^, using the complete Ensembl 85 transcriptome. Salmon was used with fragment GC bias correction, 100 bootstrap samples and using an auxiliary k-mer hash over k-mers of length 31.

### Correction of batch effects, normalisation and differential expression

RNA-Seq normalisation and differential expression were performed using DESeq2, version 1.18.1^40^. Transcript read counts were summarised to per-gene counts as the total in all transcripts of that gene (based on the transcriptome table created in Salmon). For MOFA analysis and visualization, read counts were rlog transformed (blind = True) using the rlog function from DESeq2. Samples were adjusted for sequencing batch accounting for PC and MM subgroup effects using the removeBatchEffects function from limma version 3.34.9^41^.

For pan myeloma differential expression analysis and inTAD analysis, the read counts for all primary myeloma samples (all 28 primary MM samples: marked MM in Supplementary Data 1 column *Disease* not including *cell lines)* were assigned to condition *disease* and the ND samples (5, excluding ND3-: marked Supplementary Data 1 N/A in the *Disease* column*)* were assigned to condition *normal*. Counts for CD19^+ ^and CD19^−^ samples from the same NPC donors were collapsed using collapseReplicates from the Deseq2.

To identify genes with significantly different expression between normal and cancer PCs, a Wald test was performed using DESeq2, on all samples, comparing cancer to normal, accounting for sequencing batch. Results were designated significant if they had an adjusted *p*-value (*p*-adj) <0.1 and absolute log2FoldChange greater or equal to 1.5 between cancer and normal (pan-myeloma genes).

For the subgroup differential expression analysis, the read counts for all primary samples with cytogenetic information were used (5 ND and 23 primary MM samples; O1, 2, 3, 4 and 5 and cell lines samples, labelled as MMCL were not used, Supplementary Data 1). The assigned subgroup for each sample is shown in Supplementary Data 1*(*MAF, MMSET, CCND1, HD or ND PC: *N/A* values). Similar to the pan myeloma differential expression analysis, gene counts for CD19 ^+ ^and CD19^−^ samples from the same NPC donors were collapsed using collapseReplicates. To identify genes where expression significantly varied among subgroups, a Log Ratio Test (LRT) of subgroup MM vs. PC (condition) accounting for batch was performed using DESeq2. Results were designated significant if they had an adjusted *p*-value (*p*-adj) <0.1 and absolute log2FoldChange ≥1.5, either between at least one MM subgroup and normal (subgroup genes).

Annotated and unannotated Transcription Start Sites (TSS) —In order to obtain unannotated TSS present in the PC and MM samples (primary and cell lines), RNA-seq reads were mapped using Hisat v0.1.6^42^. Stringtie version 1.2.3^43^ was used to assemble mapped reads in conjunction with the reference geneset to generate novel transcripts. Single exon transcripts were removed from this list (under the possibility that they may be eRNA transcripts). The TSS of these novel transcripts were taken to be the first 1 bp of the transcript. Annotated TSS were obtained by taking the first 1 bp of coding and non-coding genes from Ensembl v85.

Annotated and unannotated TSS sites were transformed into promoter regions by extending 2 kb upstream of the TSS, and 100 bp downstream to cover the TSS site using Bedtools version 2.22.1^44^; these are referred to as promoter sites.

### ATAC-seq analysis

A general overview of the analysis steps is illustrated in Supplementary Fig. 8.

Read cleaning and filtering—Raw paired-end ATAC-seq reads were quality controlled using FastQC. ATAC-seq adapters were removed, uncalled bases (N’s) on ends of reads were trimmed using Cutadapt version 1.9.1. Only read pairs with both single end fragments remaining were kept. A second quality pass was performed using Sickle version 1.33 (https://github.com/najoshi/sickle), trimming was performed with a sliding window average Phred quality threshold of 30 (without five prime trimming). Only pairs with a minimum of 20 bases in each read were retained.

Mapping and calling chromatin accessible peaks—As per the ENCODE ATAC-seq pipeline, the remaining paired-end reads were mapped using Bowtie2 version 2.3.0^45^ in paired-end mode to the human genome reporting up to 4 alignments per read, with maximum fragment length 2000 bp.

Processing was performed using pipeline_ATAC_consensus_balanced_peaks (https://github.com/jaime11/pipeline_atac_consensus_balanced_peaks) based on the ENCODE pipeline and recommendations for ATAC-seq processing (https://www.encodeproject.org/atac-seq/) to produce peaks in signals and reads in peaks per sample, using the CGAT-core framework.

Briefly, the pipeline first filters correctly mapped reads. Reads removed using Samtools version 1.3.1)^46^ include: unmapped (read pair or one of the reads), reads failing platform, orphan reads (one of the reads in the pair removed), read pairs mapping to different chromosomes (-F 524 -f 2 Samtools flags). Read pairs non-overlapping reads in RF orientation were removed with Samtools and Bedtools and multimapped reads using the assign_multimapper script from https://github.com/kundajelab/bds_pipeline_modules/blob/master/utils/assign_multimappers.py. Duplicates were marked using Picard Markduplicates version 1.135 (https://broadinstitute.github.io/picard/) and duplicates removed using Samtools.

Read pairs were converted into two single end tags and Tags in mitochondrial and non-standard chromosomes were removed. Any bases soft clipped in mapping were re-added and the TN5 added base pairs on the 5′ sites of each read were trimmed (these are referred to as shifted tags).

We created two consensus peak sets: the pan myeloma set and the subgroup set. For the pan myeloma set (Supplementary Fig. 8a), all primary samples (28 primary MM samples 5 ND samples, excluded cell line samples and ND3-, Supplementary Data 1) were used. The tags in each sample were down-sampled so that each sample had the same number of tags; samples were then merged into two categories—MM and NPC. This solves the issue of using the reads twice^47^: consensus peaks were called by first pooling group sample reads and then calling peaks instead of calling sample peaks and then merging.

Broad and narrow peaks were called on each pool using MACS2 version 2.1.1.20160309 (https://github.com/taoliu/MACS) with the following options for narrow peaks:

-g hs -q 0.01 --nomodel --shift -100 --extsize 200 -B --SPMR --keep-dup all --call-summits

And for broad peaks:

-g hs -q 0.01 --nomodel --shift -100 --extsize 200 --broad --broad-cutoff 0.01 --keep-dup all

Narrow and broad peaks were filtered to remove areas of low mappability (Hoffman, Ernst et al. 2013), downloaded from http://hgdownload.cse.ucsc.edu/goldenPath/hg19/encodeDCC/wgEncodeMapability/wgEncodeDukeMapabilityRegionsExcludable.bed.gz and “lifted over” from hg19 to hg38 (http://genome.ucsc.edu/cgi-bin/hgLiftOver). Peaks in ENCODE blacklist regions^48^ were also removed, downloaded from https://www.encodeproject.org/files/ENCFF419RSJ/@@download/ENCFF419RSJ.bed.gz).

The narrow peaks and broad peaks from each category (MM and ND), and peaks <200 base pairs apart were merge using bedtools.

For the subgroup consensus peak set (Supplementary Fig. 8b), the samples used were only those primary samples with cytogenetics information (the 5 ND samples except ND3- and the 23 primary MM samples in Supplementary Data 1 having *Disease* not equal to *MMCL* and also excluding samples O1, O2, O3, O4 and O5). These samples were downsampled to the minimum tags per sample. Tags from samples in the same cytogenetic group (Supplementary Data 1*Cytogenetics* groups: MAF, MMSET, CCND1, HD or ND PC: *N/A* values) were pooled into separate pools and peaks called on each pool as described above. The peaks for each pool were merged as previously using bedtools.

Sample assigned fraction—The sample assigned fraction reflects the proportion of the total reads that are mapped to areas of high accessibility compared with background noise. To calculate it, sample shifted tags were transformed extending their 5′-end 100 bp upstream and then extending 200 bp downstream (by the same values as used in MACS). They were filtered to remove tags overlapping with areas of low and high mappability defined previously. The sample assigned fraction is calculated in the following way:

$$\frac{{{{{{\rm{Extended}}}}}}\,{{{{{\rm{shifted}}}}}}\,{{{{{\rm{tags}}}}}}\,{{{{{\rm{overlapping}}}}}}\,{{{{{\rm{merged}}}}}}\,{{{{{\rm{sample}}}}}}\,{{{{{\rm{peaks}}}}}}}{{{{{{\rm{Total}}}}}}\,{{{{{\rm{extended}}}}}}\,{{{{{\rm{shifted}}}}}}\,{{{{{\rm{tags}}}}}}}$$

Annotations of the consensus peak regions—To annotate regions, the R library Annotatr version 1.8.0^49^ was used, with hg38 annotations from the library TxDb.Hsapiens.UCSC.hg38.knownGene (https://bioconductor.org/packages/3.9/data/annotation/html/TxDb.Hsapiens.UCSC.hg38.knownGene.html). Any region can overlap multiple different types of genomic annotations on both strands but for each region, a particular type is only reported once. In addition to the Peak sets described above, we also obtained annotation for a randomize sample of regions using the function randomize_regions from the R library Annotatr version 1.8.0^49^. The sample is taken without allowing overlaps and with per-chromosome regions being maintained (non-alternative, random, unknown and mitochondrial chromosomes not used).

Quantitation, normalization and differentially accessible peaks—Shifted tags from each sample were transformed to obtain only the initial 1 bp (5′ end reflecting the TN5 DNA cleavage site) and filtered to remove tags overlapping with areas of low and high mappability defined previously. Tags were overlapped with the two peak sets (pan-myeloma peaks and subtype peaks) using ‘bedtools intersect’ to generate tag counts in each sample for each of the two peak sets (Supplementary Fig. 8). The different tag counts in each peak set serve as the input data and are specified in each analysis type.

To account for batch effects, each sample was assigned to a batch based on sequencing run and sample preparation date. All samples from a batch that contained only a single sample passing quality control were assigned to a single batch and used as a reference level for batch effect removal (Supplementary Fig. 8).

For MOFA analysis, inTAD analysis and visualization, the tag counts in pan-myeloma peaks from all primary samples (all 28 primary MM samples and and the 5 ND samples excluding ND3-, Supplementary Data 1) were used. Tag counts were rlog transformed and normalized using the rlog function from DESeq and batch effects removed accounting for PC and MM subgroup effect (placing samples O1, O2, O3, O4 and O5 assigned to the *Unknown* MM subgroup and ND1+, ND1−, ND2+, ND2−, ND3+ to the *ND* subgroup, Supplementary Data 1) using the removeBatchEffect function from limma.

To verify the findings for the primary samples in our study in samples from derived from cell lines and from Jin et al.^11^, we created rlog transformed and normalized matrices following the same procedure as above for our primary samples (using the pan myeloma peak set generated from our primary samples), up to the batch correction step. We then combined the new matrix with our primary samples and performed batch correction using a version of removeBatchEffects modified to use dummy contrast encoding rather than simple contrast encoding, such that our primary samples were not altered by the process and the other samples were corrected to match them. As the choice of reference point is arbitrary (grand average in simple contrast, and reference sample mean in dummy contrast), the only difference this change will make is to hold our samples constant. The relationship between samples is maintained in either contrast encoding scheme.

To identify differentially accessible peaks between normal and cancer samples, (referred to as pan myeloma differentially accessible peaks), the tag counts in pan myeloma peak from all primary samples were used. The 28 primary MM samples were assigned to the disease condition, and the 5 ND samples were assigned to the healthy condition (Supplementary Data 1, Supplementary Fig. 8a). The MMCL cell lines, and the failed sample ND3- were excluded. Read counts for the pan myeloma peaks were used. Read counts for CD19^+ ^and CD19^−^ samples from the same NPC donors were collapsed using collapseReplicates. Significance was determined by Wald test using DESeq2 and the design formula ~ batch + condition. Outlier removal using Cook’s distance was disabled. Regions were designated as significantly changed if they had a Benjamini-Hochberg corrected p-value of <0.1 and a log_2_ fold change of at least 1 between cancer and normal.

To identify peaks differentially accessible between subtypes (referred to as subgroup differentially accessible peaks from here on), the tag counts in the subtype peaks set from all primary samples with cytogenetics information (the 5 ND samples and 23 primary MM samples. *MMCL* cell line, failed sample ND3- and the samples O1, O2, O3, O4 and O5 were excluded, Supplementary Data 1). Samples were assigned to one of the conditions MAF, MMSET, CCND1, HD or ND PC (Supplementary Fig. 8b) based on the cytogenetic information in Supplementary Data 1. Read counts for CD19^+ ^and CD19^−^ samples from the same NPC donors were collapsed using collapseReplicates. To determine regions that differed between subtypes and the ND PC samples, a differential analysis was performed using a Log Ratio Test accounting for batch with the design ~ batch + subtype compared to the reduced model ~ batch. Significant regions were those having adjusted *p*-value (*p*-adj) <0.1 and absolute log_2_FoldChange > 1 for any MM subgroup vs. NPC comparison.

### Characterising candidate enhancers and regulated genes

To obtain myeloma-differential enhancers, we took the set of DAR that were differentially accessible in at least one subtype compared to NPC (subgroup differentially accessible peaks) and removed any regions that overlapped the unannotated and annotated promoter site using Bedtools.

We associated these significantly different accessible regions with genes within 1 Mb that had significantly differential expression in the appropriate subtype.

### Enrichment analysis

Enrichment analysis was carried out using gene sets from either MSigDB or Enrichr. MSigDB gene category membership was obtained from the msigdbr package^50^. Enrichr gene-category relations were obtained using the Enrichr API^51^. Gene set enrichment was calculated using the goseq R package version 1.36.0, correcting for gene length. p-values for over-representation were corrected using the Benjimini-Hochberg procedure. For enrichment of DEG associated with DAR, either all genes not filtered by DESeq, or only DEG were used as a background as noted in the text.

### Correlation of gene-expression with peak accessibility in the same TAD

In order to measure the correlation of gene expression with peak accessibility in the same TAD, InTAD version 1.2.3 was used^14^. As input, the rlog transformed read count values of pan myeloma DEG and pan myeloma differentially accessible peaks (pan-myeloma analysis). The maximum distance allowed between the peaks and genes was 1 Mb. TAD locations from the cell line GM12878^13^ were used. Peaks and genes were correlated using the Pearson correlation coefficient and p-values were corrected using the findCorrelation method from the inTAD package (adj.pval parameter set to TRUE). 1733 inTAD correlations were obtained between pan myeloma differentially accessible peaks and protein-coding pan myeloma DEG.

To measure the significance of the bias towards positive correlation between DARs and DEGs within the same TAD we performed a permutation test. We randomly permuted the DAR-DEG pairs 1000 times. For each permutation we calculated the mean correlation for each random DAR-DEG pair and also the fraction of such associations deemed significant at *q* < 0.05 by InTAD. We compared this null distribution to the observed mean of correlation coefficients where DEG and DAR were paired within TADs. In all cases both the mean correlation coefficient, and the fraction of associations deemed significant were lower in the random permutations than in the observed set.

To test if this effect was limited to pairs within the same TAD, or was purely dependent on the distance between gene-region pairs, we divided all DAR-DEG pairs within 1MB into 10 bins (of 100 kb distance). Within each distance bin we picked an identical number of DAR-DEG pairs that were in the same TAD (in-TAD) and that were not (out-TAD) to generate distance matched sets of DAR-DEG pairs either in or not in the same TAD. We then compared the average correlation of in-TAD and out-TAD DAR-DEG pairs using a Mann–Whitney *U* test. We repeated this process 100 times. In all 100 sets the correlation for pairs within TADs was significantly higher than those of distance matched pairs not in the same TAD (*p* < 0.05). We report the average *p*-value across these 100 matched sets.

To identify likely target genes of ATAC regions selected for MOFA analysis, a similar process was followed, except that the 5000 ATAC regions were compared to all expression from all genes in the same TAD (not just those that were differentially expressed).

### Modelling effect of differential promoters and candidate enhancers on gene expression

Differentially regulated promoters were obtained by intersecting the TSS set described above with the pan myeloma differentially accessible peaks using bedtools intersect. Since only one differentially accessible peak was significantly more open in healthy samples than MM samples according to our log_2_FoldChage > 1 and *q* value <0.1 criteria, differentially accessible peaks were compared to upregulated genes rather than differentially regulated genes. The number of peaks of increased accessibility within 500 kb of the TSSs of differential upregulated genes was calculated by taking pan myeloma differentially accessible peaks, after removal of TSSs, removing the single peak more open in normal PC, and then counting overlaps with the TSS regions around each gene extended by 500,000 nt in each direction, again using bedtools intersect. Logistic regression was performed to predict significant upregulation of a gene using the presence of increased accessibility at the promoter and the count of other peaks of increased accessibility within 500 kb using R function glm, with a binomial family and logit link function.

### Multi-omics factor analysis (MOFA)

MOFA inputs—For ATAC-seq, the rLog transformed, normalized and batch corrected tag count for the pan-myeloma peak set were obtained for all samples (described above in the section *Quantitation, normalization* and *differentially accessible peaks*). Regions corresponding to sex chromosomes (chrX and chrY) and regions overlapping multi-exon TSS (see above) were removed. This yielded 273,216 remaining regions. The overall variance per peak (across the transformed counts for all 28 primary MM samples the 5 ND samples, excluding MMCL cell lines and ND3-, Supplementary Data 1) was calculated and only the 5000 peaks with the highest variance were selected.

For RNA-seq, the rLog transformed, normalized and batch corrected RNA-seq read counts for all samples were obtained (described above). Genes on sex chromosomes were removed from the table. The variance per gene was calculated and only the 5000 genes with the highest variance again using all primary patient samples.

Training—Using the R library MOFAtools version 0.99.0^52^, the ATAC-seq and RNA-seq tables were input to MOFA. The default data and model options were used and “Gaussian” data was selected both for ATAC-seq and RNA-seq. The training options used that were different to the defaults were: dropping factor threshold of 0.01, 10,000 maximum iterations, minimum tolerance convergence threshold of 0.01. Information on each samples’ subgroup (cytogenetic MM subgroup, PC or “MM_OTHER” for MM samples with no cytogenetic information), condition (PC or MM) was not used by MOFA but was included for the analysis of the results. The variance explained was divided into 17 LF.

The R script used to train the MOFA model can be found in:

scripts/train_MOFA.R

Models using different random initializations were trained and fit using only RNA-seq data, or both RNA-seq and ATAC data.

Silhouette score for samples—For each model, each sample was assigned a label determined by its cytogenetic subgroup (including unknown cytogenetic samples, considered to be in the same group), the per sample silhouette score was obtained by calculating Euclidean distances between samples either all LFs as dimensions or only LF1 to LF5. This was done using the “silhouette” function from the “cluster” R package version 2.0.6. The mean silhouette score for each subgroup and model is shown.

Projection of test samples—MOFA decomposes the features matrix Y into a loadings matrix W and a factors matrix Z (Y = W∗Z). To project new samples onto the same LF space we require a rotation matrix W’ such that Y∗W’ = Z. We estimated this by computing the generalized inverse of the MOFA loadings matrix using the ‘ginv’ function from the matlib package. When the resulting matrix was multiplied by the features matrix it gave the same loadings for the primary samples correct to a linear factor.

### Classification of samples using large cohort datasets

Additional molecular classification of samples used in this study was performed using previously identified, “gold standard” MM subgroup classifiers (Arkansas study, *n* = 414 patients^12^). For this purpose, primary MM WGS and RNA-seq data from 892 patients were obtained from the MMRF CoMMpass study online portal (https://research.themmrf.org/). In addition, clinical annotation files containing the subgroup stratification of each sample based on SeqFISH genetic translocation analysis (WGS-based) and primary oncogenic markers overexpression calls (RNA-seq) were obtained from the same repository. Correlation analysis of samples used in this study and the MMRF cohort was performed based on the expression of the “gold standard” MM subgroup classifiers^12^, using *hclust* (method: Pearson, linkage: complete) and visualized using *pheatmap*2 R packages.

Measuring discrimination of subtypes—For both the ATAC and RNA-seq combined model and the RNA-seq only model, we calculated Fisher’s Discriminant Ratio for each LF as:

$$S(X)=\frac{{\sigma }_{{{{{\mathrm{between}}}}}}^{2}}{{\sigma }_{{{{{\mathrm{within}}}}}}^{2}}=\frac{{V}_{b}}{{V}_{w}}$$

where ${V}_{b}$ the between groups variance of *X* and ${V}_{w}$ the within-group variance of *X* are calculated as:

$${V}_{b}=\frac{1}{N-G}\mathop{\sum }\limits_{g=1}^{G}{n}_{g}{(\overline{{x}_{g}}-\overline{\bar{x}})}^{2}$$

$${V}_{w}=\frac{1}{N-G}\mathop{\sum }\limits_{g=1}^{G}({n}_{g}-1){s}_{g}^{2}$$

where *G* is the number of subtypes, *N* the total number of samples and ${n}_{g},\,\overline{{x}_{g}},{s}_{g}^{2}$ are the number of samples, the mean and the variance of subgroup *g* respectively and $\overline{\overline{x}}$ is the grand mean. For each model we selected the LF that gave the greatest discriminant ratio. Linear discriminant analysis was performed on the first 5 LFs of each model using the ‘lda‘ function from the R MASS package.

### Chromatin state analysis

The Chromatin State Segmentations (12 states) by ChromHMM for the 173 cell types for the GRCh38 genome available to date in The DeepBlue Epigenomic Data Server (https://deepblue.mpi-inf.mpg.de/) were retrieved. Only cell types from the B-cell lineage were kept, in total, 19 samples.

For the chromatin state heatmap, 1733 region—gene interactions were calculated using inTAD (see *Correlation of gene-expression with peak accessibility in the same TAD* section). Of these 1190 interactions were significant (adj.*p*val < 0.05), from 931 unique regions (i.e. some regions correlated with multiple genes). These pan-MM enhancers were divided into 200 bp windows. The chromatin state segmentations are already divided into the same 200 bp windows. Each 200 bp enhancer region was intersected with the cell states table to obtain the chromatin state for each cell type in that region using Bedtools intersect. For each candidate enhancer, a “predominant” state was calculated (i.e. the state which accounts for the largest number of 200 bp windows). Enhancers were filtered, keeping only those where a predominant state was found in 15/19 cell chromHMM samples. For 99 of the 931 regions there was no predominant chromatin state in 16 or more samples leaving 832 useable candidate enhancer regions. The matrix of predominant states was hierarchically clustered using the Gower metric with the daisy function from the “cluster” R package using Ward.D2 linkage (where a candidate enhancer had no predominant state in a given cell line, this was considered missing data). This clustering of the predominant states was used to determine the order of regions, and therefore order the matrix of 200 bp windows, leaving 200 bp windows from the same region together, but regions ordered according to the clustering.

The 832 potential enhancers with a predominant state were classified using their predominant states. States 7 (Genetic Enhancer High), 9 (Active Enhancer High), 10 (Distal Active Promoter 2 kb High) and 12 (Active TSS High Signal H3K4me3 H3K27Ac) were classified as enhancer states. The final two were classified as enhancers as have removed annotated and unannotated TSS and it has recently been suggested that H3K4me1 is not a requirement for enhancer driven transcription in mESC^23^ and in Drosophila melanogaster^53^ and that highly active enhancers are marked by H3K4me3 and not H3K4me1 in flies and mESCs^54^. Regions were classified on the following criteria:

Regions were first divided into two classes based on B-cell states (all cell types except PC, MM, MM cell lines) 12/19 cell types into:

- Category 1: Deactivated enhancers in B-cells: 3 or less out of 12 B cell types having an active enhancer state.
- Category 2: B-cell activated enhancers: B cell activated enhancers: 4 + B cell types having an active enhancer state.

Category 1 (deactivated in B-cells) was then subdivided into:

- De novo MM enhancers: 0/2 PC cells and 2 + (2 or more)/4 MM having an active enhancer state.
- All cell types deactivated regions/no predominant state: 0/2 PC cells and 1 or 0/4 MM.
- PC enhancers, deactivated in MM and Bcell: 1 + /2 PC and 1 or less/4 MM.
- PC and MM only enhancers: 1 + /2 PC and 2 + /4 MM.

Category 2 (generally activated B cell) was subdivided into:

- MM only reactivated B cell enhancers: 0/2 PC cells and 2 + /4 MM having an active enhancer state.
- PC and MM deactivated regions/no predominant state: 0/2 PC cells and 1 or 0/4 MM.
- Deactivated MM, PC and B cell enhancers: 1 + /2 PC and 1 or 0/4 MM.
- PC, MM, B-cell enhancers: 1 + /2 PC and 2 + /4 MM.

For the analysis of CLL samples, representative samples for ATAC-seq and chromatin state were chosen for naïve, memory and germinal B-cells, tonsillar PC and CLL samples, and ATAC and ChromHMM tracks downloaded from http://inb-cg.bsc.es/hcli/IDIBAPS_Biomedical_Epigenomics/CLL_Reference_Epigenome/. ATAC traces were created by averaging signal for each sample. To display chromatin state tracks, the emissions matrix from^36^ and Blueprint Epigenomics were manually inspected. The 12 states, although named differently matched almost exactly, and the states from^36^ were displayed using same colour as the colour used for the closest matching state from Blueprint.

### Inference of gene regulatory networks

Digital footprinting analysis was performed using existing methodologies with minor modifications^55,56^. Mapped reads files (bam files) were sub-sampled to the minimum depth across all samples and merged based on their corresponding cytogenetic subgroup (ND, MMSET, MAF, HD, CCND1). Consensus chromatin accessibility regions were obtained by merging open ATAC-seq peak files from individual samples for each subgroup. The Wellington_footprints.py command from the pyDNAse/Wellington^55^ package was used to obtain footprints on the consensus ATACseq regions for each subgroup, using the parameters: *-fdr 0.05 -fdrlimit -10 -A*. Predicted binding maps for a set of 769 highly curated human TF motifs from the HOCOMOCOv1^57^ collection on identified footprints were generated for each subgroup using the *findMotifsGenome.pl* command of the Homer_v4.9 package^58^ and annotated to target genes using the ChIPpeakAnno R package. TF motif occurrences were also calculated for the consensus accessible chromatin regions for each subgroup and used as background. Only TFs with expression of TPM ≥ 10 in at least one sample within each subgroup were considered for downstream analysis. In each subgroup, the adjusted relative frequency for each TF, t was calculated as:

$${{{{{\mathrm{Rel.}}}}}}\,{{{{{\mathrm{fre{q}}}}}}}_{t}=\frac{\frac{{m}_{t}}{n}}{\frac{{M}_{t}}{N}}$$

where

*m*_*t*_ is the number of motif occurrences in *n* total number of footprints

*M*_*t*_ is the number of motif occurrences in *N* total number of consensus ATAC-seq regions.

Footprint plots were generated with the dnase_average_profile.py command from the pyDNAse/Wellington package, using the ATACseq mode (-A). TF network visualization for each MM subgroup network was performed using Cytoscape 3.5 by weighing node size based on TF gene expression and node colour based on the adjusted relative frequency score. The NetworkAnalyzer tool, built in Cytoscape3.5 software, was used for network metrics analysis for each MM subgroup network. Auto-regulatory TF loops were defined as the cases where TFs are predicted to bind to and regulate their own gene.

### Differential DNA footprinting on *CCND2*^*high*^ versus *CCND2*^*low*^ HD samples

Differential footprinting was performed in patients with HD myeloma with high (*n* = 4) and low (*n* = 4) *CCND2* mRNA expression (mean normalized count 0.23 vs 138.9, *p* =  0.016). Bam files were sub-sampled to the minimum depth across eight samples and merged according to their *CCND2* levels (high, low). Consensus open chromatin regions were obtained by merging the ATAC-seq peak files from each sample. The wellington_bootstrap.py command from the pyDNAse/Wellington^55,56^ package was used to obtain differential footprints on the consensus ATACseq regions for each subgroup, using the parameters: -fdr 0.05 -fdrlimit -8 -A. We considered only footprints with wellington score >10 for downstream analysis. Predicted differential binding maps for human TF motifs from the HOCOMOCOv12 collection were obtained using the *findMotifsGenome.pl* command of the Homer_v4.9 package^16^. For each TF with expression of TPM ≥ 10 in at least one sample, the differential predicted frequency was calculated for each *CCND2* super-enhancer constituent region as:

$$\varDelta {f}_{t}=\frac{{\sum }^{}({{h}_{t}}^{H}* {W}^{H})}{{\sum }^{}({{h}_{t}}^{L}* {W}^{L})}$$

where

*Δf*_*t*_ is the differential predicted frequency for transcription factor *t*

*h*_*t*_ is the Homer motif purity score on differential footprints

*W* is the Wellington differential footprint probability score

The superscripts *H* and *L* refer to CCND2^High^ and CCND2^Low^ conditions, respectively.

### Reporting summary

Further information on research design is available in the Nature Research Reporting Summary linked to this article.

## Supplementary information

Supplementary Information

Description of Additional Supplementary Files

Supplementary Data 1

Supplementary Data 2

Supplementary Data 3

Supplementary Data 4

Supplementary Data 5

Supplementary Data 6

Supplementary Data 7

Reporting Summary

Source file

## Data Availability

The data that support this study are available from the corresponding authors upon reasonable request. Raw sequencing data, peak sets and gene quantifications are deposited in the GEO database under the accession code GSE153381. Additional MM patient RNA-seq data were obtained from the MMRF CoMMpass study database (https://research.themmrf.org/rp/download?level=IA15) and EGA repository (accession number phs000748). Due to patient privacy regulations, access to MM patient RNA-seq data from the MMRF CoMMpass study database is restricted, but can be granted from the database developers at https://themmrf.org/rg-signup/. Additional data from the Jin et al.^11^ study were obtained from the EBI repository (accession number PRJEB25605). Chromatin State Segmentations (12 ChromHMM states) for 19 B-cell lineage samples were retrieved from The DeepBlue Epigenomic Data Server (https://deepblue.mpi-inf.mpg.de/). The source data are provided with this paper.

## Code availability

Custom code used in this work is available at: https://github.com/sudlab/alvarez_et_al and https://zenodo.org/badge/latestdoi/346203815
