# Supplementary material for: Chromatin-based, in cis and in trans regulatory rewiring underpins distinct oncogenic transcriptomes in multiple myeloma
Source: Nat Commun. 2021 Sep 14;12:5450. doi: 10.1038/s41467-021-25704-2 (PMC8440555; doi:10.1038/s41467-021-25704-2)
Supplement: Supplementary file 1 — Supplementary Information [file 41467_2021_25704_MOESM1_ESM.pdf]

Chromatin-based, *in cis* and *in trans* regulatory 'rewiring' underpins distinct oncogenic transcriptomes in multiple myeloma

Jaime Alvarez-Benayas, Nikolaos Trasanidis, Alexia Katsarou *et al*

Supplementary File

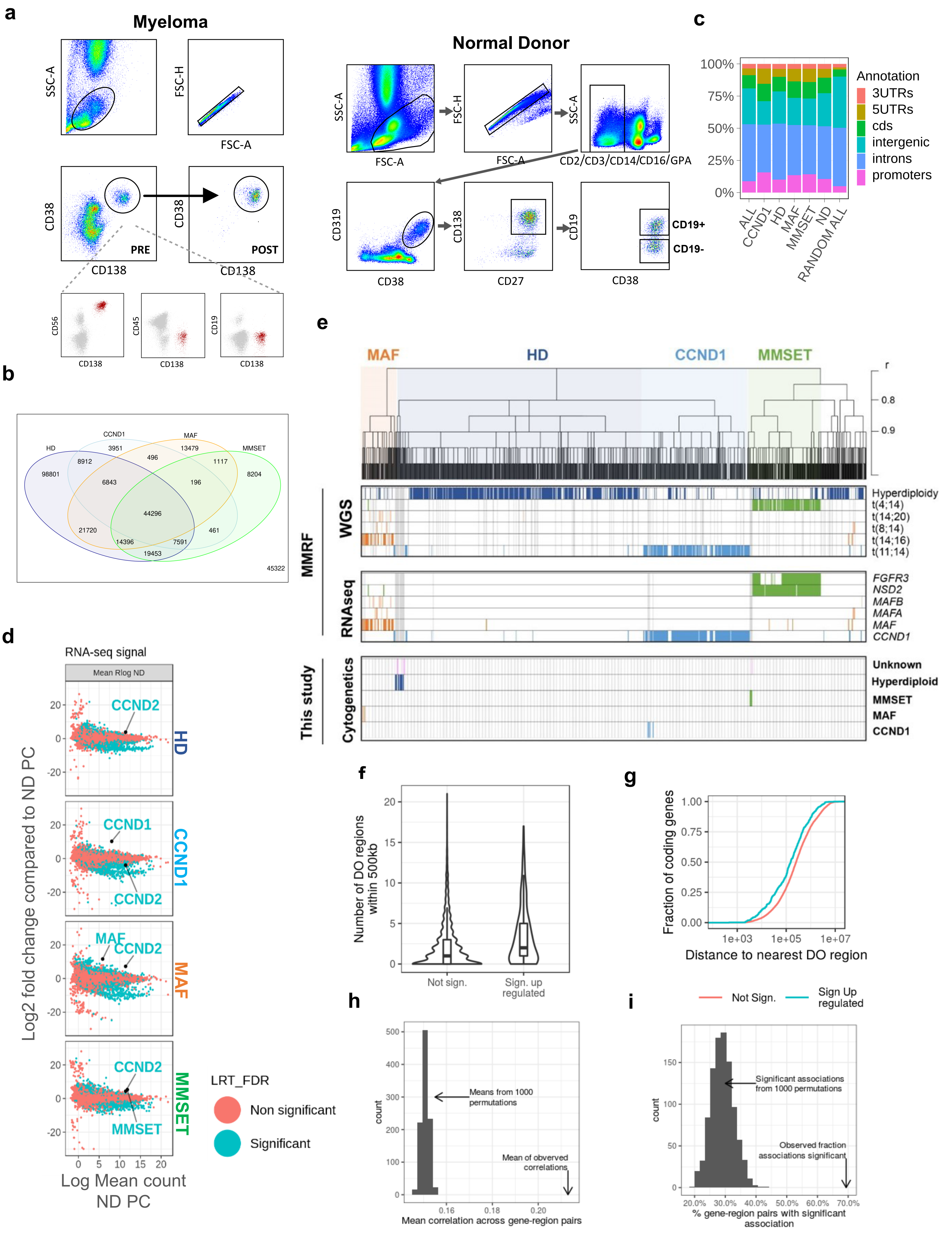

a
Myeloma
SSC-A
FSC-H
FSC-A
CD38
CD138
PRE
POST
CD56
CD45
CD19
Normal Donor
CD2/CD3/CD14/CD16/GPA
CD319
CD27
CD19+
CD19-
c
100%
75%
50%
25%
0%
ALL
CCND1
HD
MAF
MMSET
ND
RANDOM ALL
Annotation
3UTRs
5UTRs
cds
intergenic
introns
promoters
b
HD
CCND1
MAF
MMSET
98801
8912
3951
496
13479
1117
8204
6843
196
44296
21720
14396
7591
461
19453
45322
d
RNA-seq signal
Mean Rlog ND
Log2 fold change compared to ND PC
CCND2
CCND1
MAF
MMSET
Log Mean count ND PC
LRT_FDR
Non significant
Significant
e
MAF
HD
CCND1
MMSET
r
0.8
0.9
MMRF
WGS
RNAseq
This study
Cytogenetics
Hyperdiploidy
t(4;14)
t(14;20)
t(8;14)
t(14;16)
t(11;14)
FGFR3
NSD2
MAFB
MAFA
MAF
CCND1
Unknown
Hyperdiploid
MMSET
MAF
CCND1
f
Number of DO regions within 500kb
Not sign.
Sign. up regulated
g
Fraction of coding genes
1e+03
1e+05
1e+07
Distance to nearest DO region
Not Sign.
Sign Up regulated
h
count
Means from 1000 permutations
Mean of observed correlations
Mean correlation across gene-region pairs
i
Significant associations from 1000 permutations
Observed fraction associations significant
% gene-region pairs with significant association

Supplementary Figure 1 (related to Fig 1). Chromatin accessibility and transcriptome alterations detected in multiple myeloma versus normal plasma cell samples.

a) Pre- and post-immunomagnetic bead selection of myeloma bone marrow PC (left); normal donor bone marrow PC were selected using flow-sorting according to indicated markers. In two samples both $CD19^{+}$ and $CD19^{-}$ PC were obtained while in a third sample only $CD19^{-}$ PC were obtained.

b) Venn diagram showing peaks discovered in ATAC-seq from MM samples with different genetic abnormalities. The number on the bottom right corner shows the number of ND PC peaks not found in MM samples.

c) Genomic annotation of peaks discovered in samples from each subtype. Each peak may overlap more than one context. 'Random All' refers to a set of size match regions placed randomly on the genome.

d) MA plots for differential gene expression analysis for each MM subgroup against normal donor PC. Prominent oncogenes for each subgroup are indicated here.

e) Unsupervised clustering of RNA-seq profiles from MM samples used in this study (n=30) along with samples from the MMRF Compass study (n=892), based on the expression of MM genetic subgroup classifiers identified by [1]. From top to bottom: Samples clustering performed using the Pearson's correlation distance (r coefficient indicated on top right panel). Clinical annotation for MMRF Compass MM samples was based on SeqFISH genetic translocation analysis (WGS data) and over-expression of primary oncogenic markers (RNA-seq) for each subgroup. MM samples used in this study were annotated using fluorescent in-situ hybridisation (FISH) analysis.

f) Number of pan-myeloma peaks found to be significantly differentially open (DO) in myeloma samples within 500kb of genes found to be significantly upregulated in myeloma (Sign. up regulated), or not (Not sign.). Violin plots represent the distribution of scores and boxplots the 10-90 percentiles over mean.

g) Cumulative distribution function for the distance to the TSS of the nearest pan-myeloma peak found to be significantly more open in myeloma (DO, differentially open) for genes that either significantly upregulated in myeloma (Sign. Up regulated, blue) or not (Not sign., red).

h & i) Analysis of significance of DAR-DEG correlations by random permutations. Random pairing (n=1,000) of DAR-DEGs identifies lower mean correlation coefficients **(h)** and lower percentage (%) of significantly correlated DAR- DEG pairs (**i**) to be detected by chance compared to the observed values.

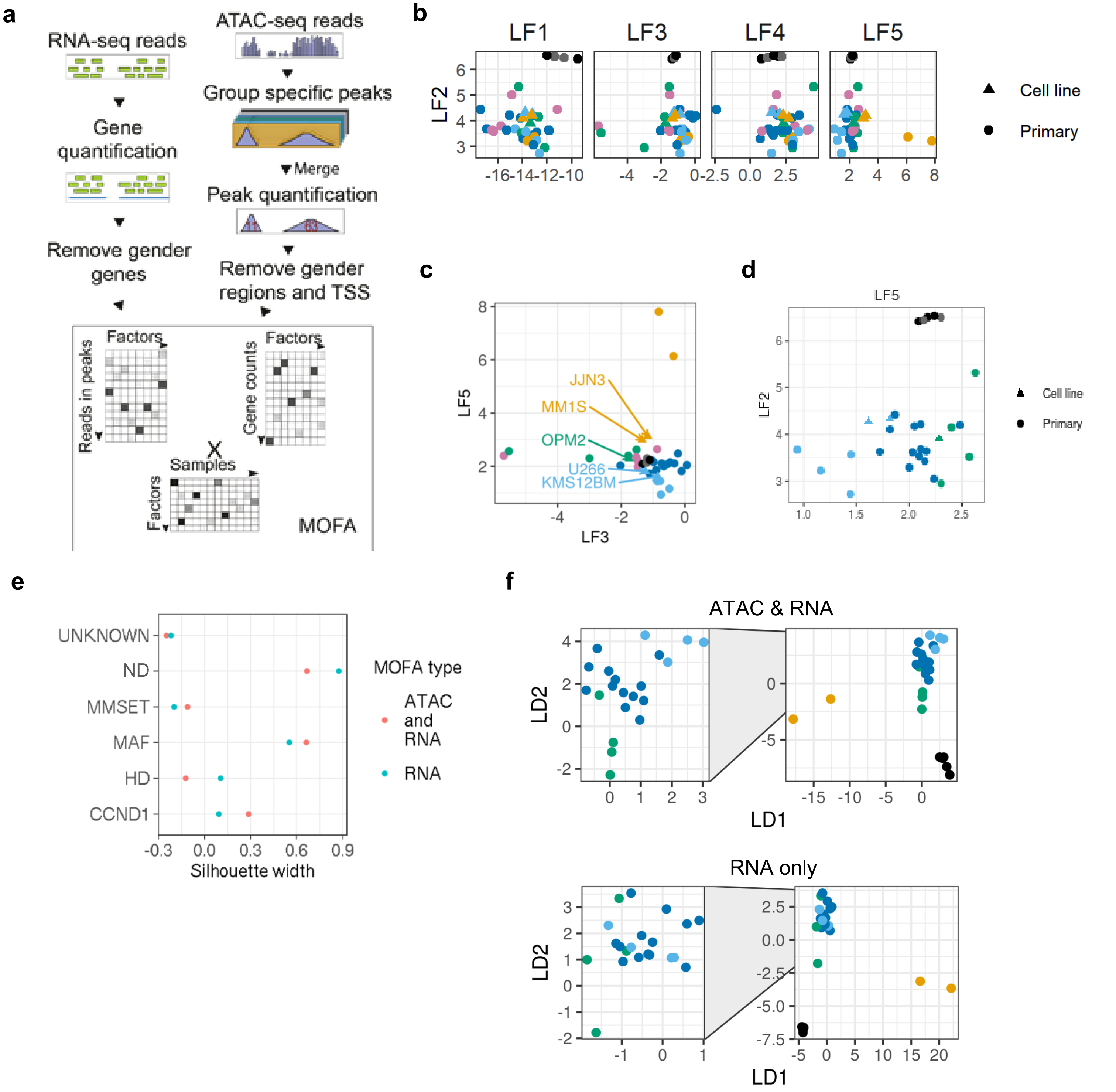
a
RNA-seq reads
Gene quantification
Remove gender genes
ATAC-seq reads
Group specific peaks
Merge
Peak quantification
Remove gender regions and TSS
Factors
Reads in peaks
Gene counts
X
Samples
Factors
MOFA
b
LF1
LF3
LF4
LF5
LF2
Cell line
Primary
c
JJN3
MM1S
OPM2
U266
KMS12BM
LF5
LF3
d
LF5
LF2
Cell line
Primary
e
UNKNOWN
ND
MMSET
MAF
HD
CCND1
Silhouette width
MOFA type
ATAC and RNA
RNA
f
ATAC & RNA
LD2
LD1
RNA only
LD2
LD1

Supplementary Figure 2 (Related to Fig 1). **Unsupervised analysis of chromatin accessibility and transcriptome profiles using MOFA.**

a) Schematic representation of MOFA analysis pipeline and steps.

b) LF scores for the first 5 latent factors resulted from combined primary and cell lines (test set) MOFA analysis model. Subtype for each sample is denoted by colour, origin is denoted by shape (primary: circle; cell line: triangle).

c) LF scores on LF5-LF3 axes from combined primary and cell lines MOFA model displays segregation of cell lines test set based on their corresponding MIE. Sample subtype is denoted by colour; origin is denoted by shape.

d) Closer snapshot of LF2-LF5 bi-plot of LF scores for combined primary and cell lines MOFA model only for CCND1, HD and MMSET subgroups

e) Comparison of MOFA models built with both ATAC-seq and RNA-seq data, or RNA-seq data only. Performance in separating subtypes is measured as the silhouette score for members of that subtype compared to all other subtypes. High silhouette score means better clustering. Distance between samples is calculated as using Euclidean distance based the first 5 latent factors.

f) Linear Discriminant Analysis performed in combined ATAC-RNA (top) and RNA-only (bottom) MOFA models displays the quality of separation of MM samples according to their corresponding cytogenetic subgroups.

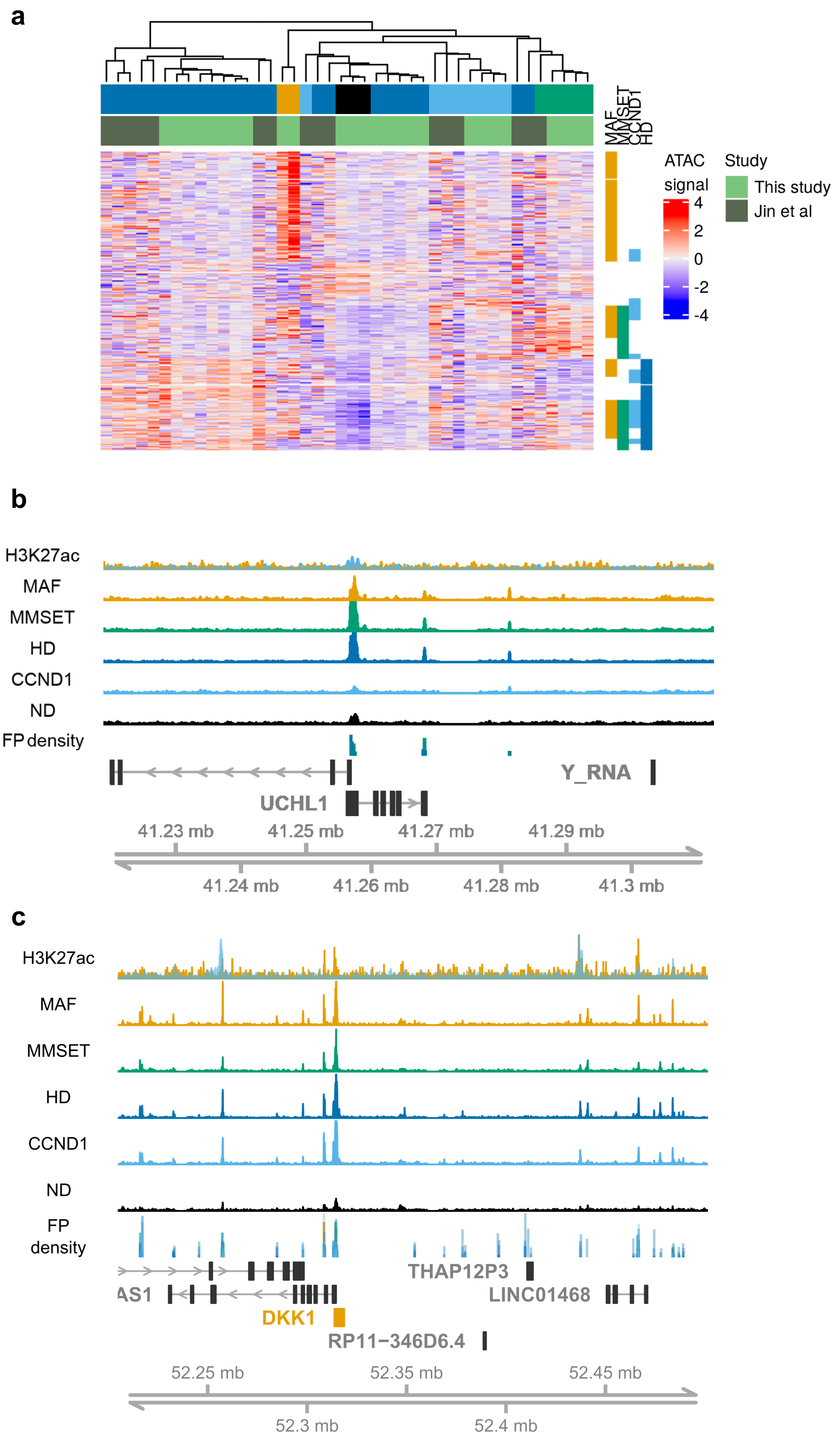
a
MAF
MMSET
CCND1
HD
ATAC signal
4
2
0
-2
-4
Study
This study
Jin et al
b
H3K27ac
MAF
MMSET
HD
CCND1
ND
FP density
Y_RNA
UCHL1
41.23 mb
41.25 mb
41.27 mb
41.29 mb
41.24 mb
41.26 mb
41.28 mb
41.3 mb
c
H3K27ac
MAF
MMSET
HD
CCND1
ND
FP density
THAP12P3
AS1
LINC01468
DKK1
RP11−346D6.4
52.25 mb
52.35 mb
52.45 mb
52.3 mb
52.4 mb

Supplementary Figure 3 (Related to Fig2). Enhanced chromatin accessibility in distal DNA elements detected in all myeloma genetic subgroups versus normal plasma cells.

a) Heatmap representation of ATAC-seq signal for all peaks found to be differentially open and within 1Mb of a significantly differentially regulated gene, as resulted from combined analysis of samples used in this study along with those from *Jin et al. 2018*. ATAC-seq signal values were z-score standardized for each row. Samples are clustered using Pearson's correlation distance. The bars on top indicate the subtype (MAF: orange, CCND1: light blue, MMSET: green, HD: blue and ND PC: black) and the source of study (light green: this study, forest green: *Jin et al 2019* study). Vertical bars to the right highlight regions where signal is >2-fold different to ND PC.

b) ATAC and H3K27ac signal around the *UCHL1* gene upregulated in MM.
Tracks show normalised ATAC-seq (named after subtype), H3K27ac signal in *MAF* translocated cell line (Orange) and *CCND1* translocated cell line (blue) and density of footprints in the ATAC-seq signal (as called by Wellington).

c) ATAC and H3K27ac signal around the *DKK1* gene, upregulated in MM**.**Tracks show normalised ATAC-seq (named after subtype), H3K27ac signal in *MAF* translocated cell line (Orange) and *CCND1* translocated cell line (blue) and density of footprints in the ATAC-seq signal (as called by Wellington).

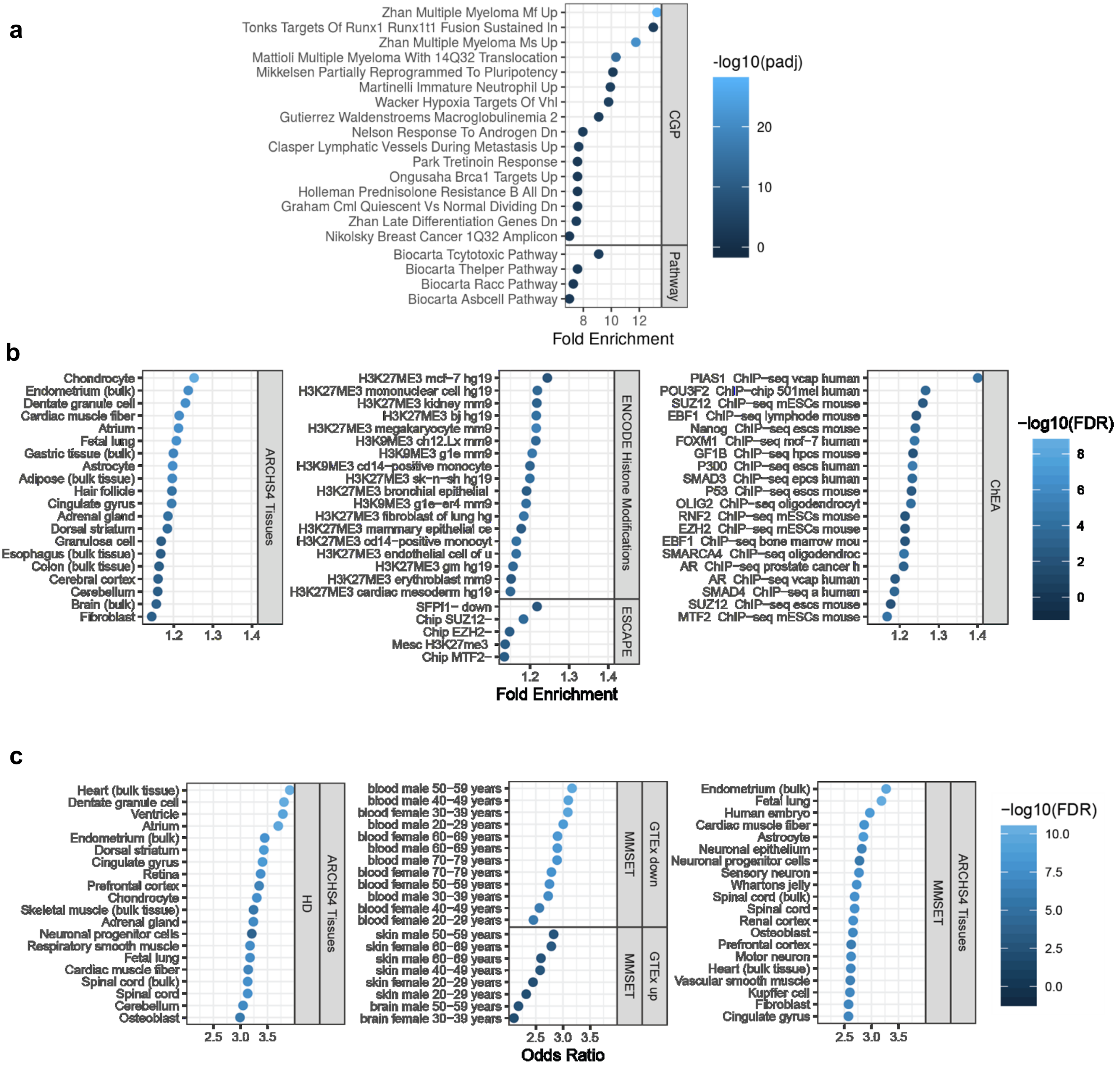
a
Zhan Multiple Myeloma Mf Up
Tonks Targets Of Runx1 Runx1t1 Fusion Sustained In
Zhan Multiple Myeloma Ms Up
Mattioli Multiple Myeloma With 14Q32 Translocation
Mikkelsen Partially Reprogrammed To Pluripotency
Martinelli Immature Neutrophil Up
Wacker Hypoxia Targets Of Vhl
Gutierrez Waldenstroems Macroglobulinemia 2
Nelson Response To Androgen Dn
Clasper Lymphatic Vessels During Metastasis Up
Park Tretinoin Response
Ongusaha Brca1 Targets Up
Holleman Prednisolone Resistance B All Dn
Graham Cml Quiescent Vs Normal Dividing Dn
Zhan Late Differentiation Genes Dn
Nikolsky Breast Cancer 1Q32 Amplicon
Biocarta Tcytotoxic Pathway
Biocarta Thelper Pathway
Biocarta Racc Pathway
Biocarta Asbcell Pathway
CGP
Pathway
Fold Enrichment
-log10(padj)
b
ARCHS4 Tissues
ENCODE Histone Modifications
ESCAPE
ChEA
Fold Enrichment
−log10(FDR)
c
HD
ARCHS4 Tissues
MMSET
GTEx down
GTEx up
Odds Ratio
−log10(FDR)

Supplementary Figure 4 (Related to Fig 2). Overview of enrichment analysis for upregulated genes within 1Mb of a non-TSS ATAC-seq peak of increased accessibility.

a) Upregulated genes within 1Mb of putative enhancers compared with all genes using the MSigDB curated pathways gene sets; CGP-Chemical/Genetic Perturbation, Pathway – Biocarta, KEGG, Reactome and Wikipathways.

b) The same genes compared to only upregulated genes using a selection of gene-set categories from Enrichr.

c) Genes upregulated in specific myeloma subgroups within 1Mb of putative enhancers from the same subgroup compared to upregulated genes using a selection of gene-set categories from Enrichr. Subgroup shown in the sidebar.

All enrichments BH corrected p-value < 0.01. A maximum of 20 gene sets is shown in any one category.

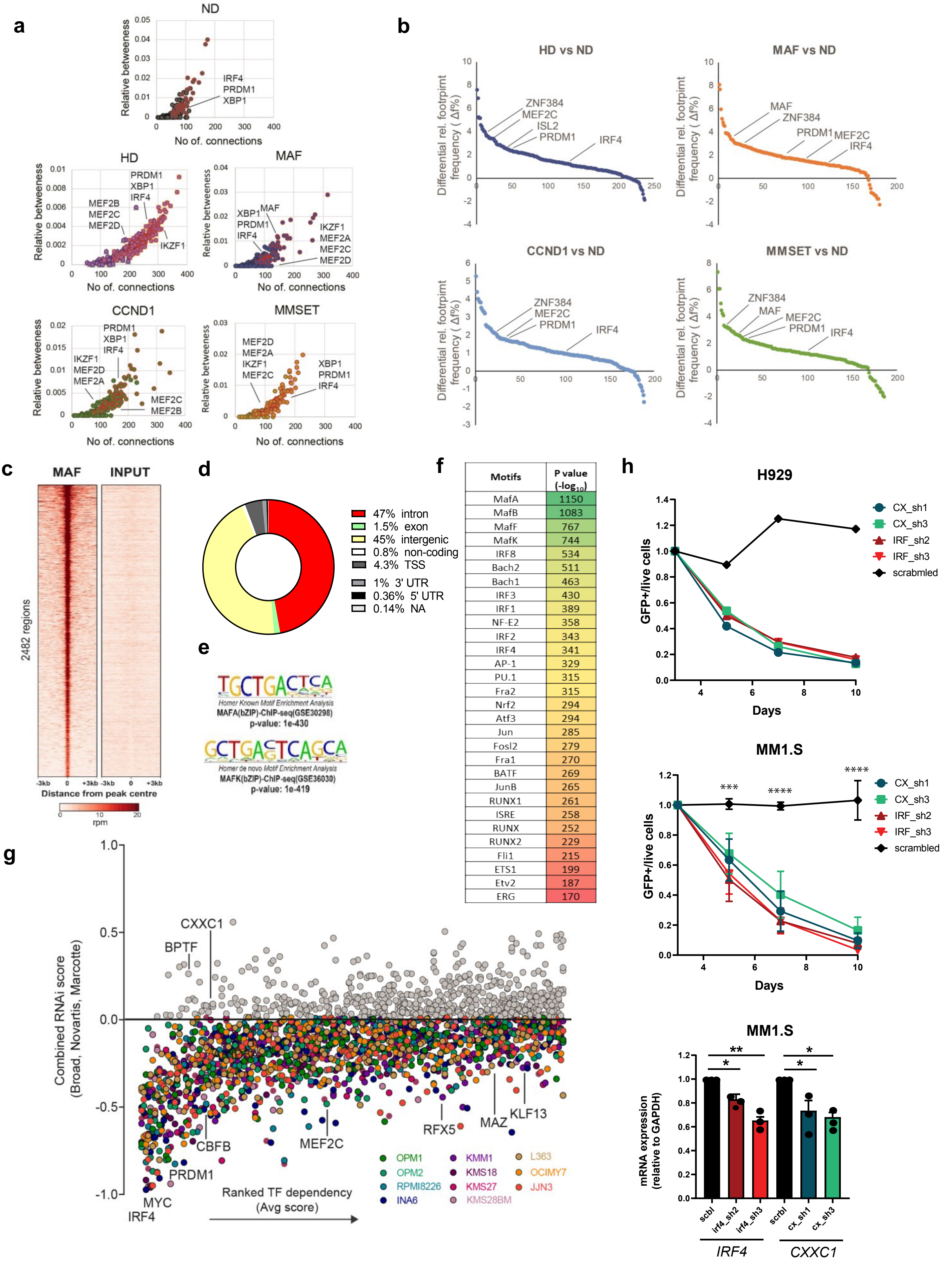

Motifs	P value (-log10)
MafA	1150
MafB	1083
MafF	767
MafK	744
IRF8	534
Bach2	511
Bach1	463
IRF3	430
IRF1	389
NF-E2	358
IRF2	343
IRF4	341
AP-1	329
PU.1	315
Fra2	315
Nrf2	294
Atf3	294
Jun	285
Fosl2	279
Fra1	270
BATF	269
JunB	265
RUNX1	261
ISRE	258
RUNX	252
RUNX2	229
Fli1	215
ETS1	199
Etv2	187
ERG	170

Supplementary Figure 5 (Related to Fig 3 & 4). Extensive transcription factor rewiring and TF dependencies with potential clinical insights detected in multiple myeloma cells.

a) Biological networks analysis depicts the number of connections and the relative betweenness centrality of each active TF across MM subgroups and normal donor PC. TFs with active auto-regulatory loops are highlighted in red outline.

b) Relative footprint frequency (Δf%) of indicated TF in myeloma subgroups versus normal donor (ND) PC.

c) Heatmap illustration of MAF-bound genomic regions in MAF-translocated MM.1S cells, as compared to input control.

d) Genomic annotation of MAF cistrome in MM.1S cells.

e) Weblogo representation of MAF motifs found to be significantly enriched in MAF-bound regions in MM.1S cells.

f) TF motif discovery in MAF-bound genomic regions in MM.1S cells.

g) TF dependency analysis using combined RNAi screens data from DepMap database (11 myeloma cell lines representing CCND1, MAF and MMSET genetic subgroups; color-coded). Established and novel TF dependencies are shown as in **Fig3d.** In this analysis, dependency is defined as combined score < -0.1 in at least 4/11 myeloma cell lines.

h) *CXXC1* and *IRF4* shRNA-mediated knockdown dependency analysis. H929 (top, n=2) and MM.1S (middle, n=4) MMCLs were transduced with scrambled shRNA, and 2 independent shRNA targeting *CXXC1* and *IRF4.* Cell viability was assessed based on the proportion of live GFP+ cells on a time-course, normalised to day3 after transduction. Knockdown *CXXC1* and *IRF4* mRNA levels of GFP+ sorted cells on day 4 after transduction on MM.1S (bottom, n=3). Error bars represent SEM. Statistical analysis was done by two-way matched-samples ANOVA test for viability (middle) and one-way matched-samples ANOVA test for mRNA (bottom) assays, followed by Tukey's post-hoc multiple correction test. N represents independent experiments. *p<0.05, ** p<0.01,*** p<0.001, **** p<0.0001

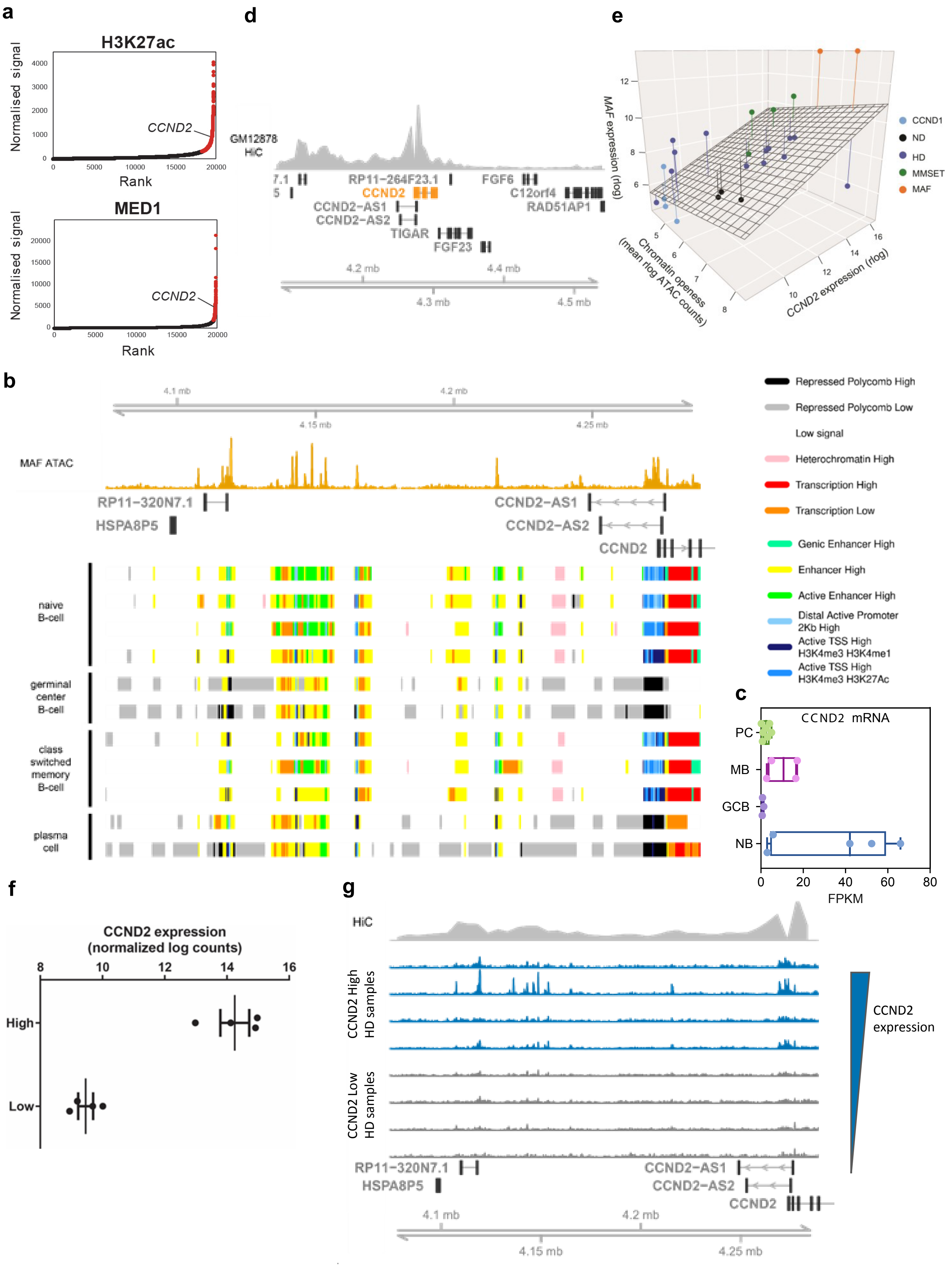
a
H3K27ac
Normalised signal
CCND2
Rank
MED1
Normalised signal
CCND2
Rank
d
GM12878
HiC
RP11-264F23.1
FGF6
CCND2
C12orf4
CCND2-AS1
RAD51AP1
CCND2-AS2
TIGAR
FGF23
4.2 mb
4.4 mb
4.3 mb
4.5 mb
e
MAF expression (rlog)
Chromatin openess (mean rlog ATAC counts)
CCND2 expression (rlog)
CCND1
ND
HD
MMSET
MAF
b
4.1 mb
4.2 mb
4.15 mb
4.25 mb
MAF ATAC
RP11-320N7.1
HSPA8P5
CCND2-AS1
CCND2-AS2
CCND2
naive B-cell
germinal center B-cell
class switched memory B-cell
plasma cell
Repressed Polycomb High
Repressed Polycomb Low
Low signal
Heterochromatin High
Transcription High
Transcription Low
Genic Enhancer High
Enhancer High
Active Enhancer High
Distal Active Promoter 2Kb High
Active TSS High H3K4me3 H3K4me1
Active TSS High H3K4me3 H3K27Ac
c
CCND2 mRNA
PC
MB
GCB
NB
FPKM
f
CCND2 expression (normalized log counts)
High
Low
g
HiC
CCND2 High HD samples
CCND2 Low HD samples
CCND2 expression
RP11-320N7.1
HSPA8P5
CCND2-AS1
CCND2-AS2
CCND2
4.1 mb
4.2 mb
4.15 mb
4.25 mb

Supplementary Figure 6 (related to Fig 5). Epigenetic characterization and developmental origins of the *CCND2* super-enhancer in multiple myeloma cells.

a) Super-enhancer (SE) calling in MM.1S MMCL using H3K27ac (top) and MED1 (bottom) ChIPseq signal.

b) The developmental origins of the *CCND2* super-enhancer as determined by ChromHMM state analysis across different B-cell types (naïve, germinal center, class-switched memory, plasma cells). ATAC-seq signal of MAF primary cells is also presented on the top panel**.**

c) *CCND2* expression in normal plasma cells (PC), memory B cells (MB), germinal center B cells (GCB) and naïve B cells (NB). Boxplots display all values as points, whisker's box (min to max) and mean expression per cell type.

d) Hi-C signal for 3D genomic interactions around *CCND2* locus with its promoter region in GM12878 B cells.

e) 3D scatter plot displaying the correlation among chromatin accessibility of *CCND2* enhancer, *CCND2* and *MAF* gene expression. The distance of each point to the linear regression fitting surface is shown as a line from the point.

f) *CCND2* RNA-seq expression levels and

g) ATAC-seq signal tracks in *CCND2*^{high} and *CCND2*^{low} patient samples within the HD molecular subgroup.

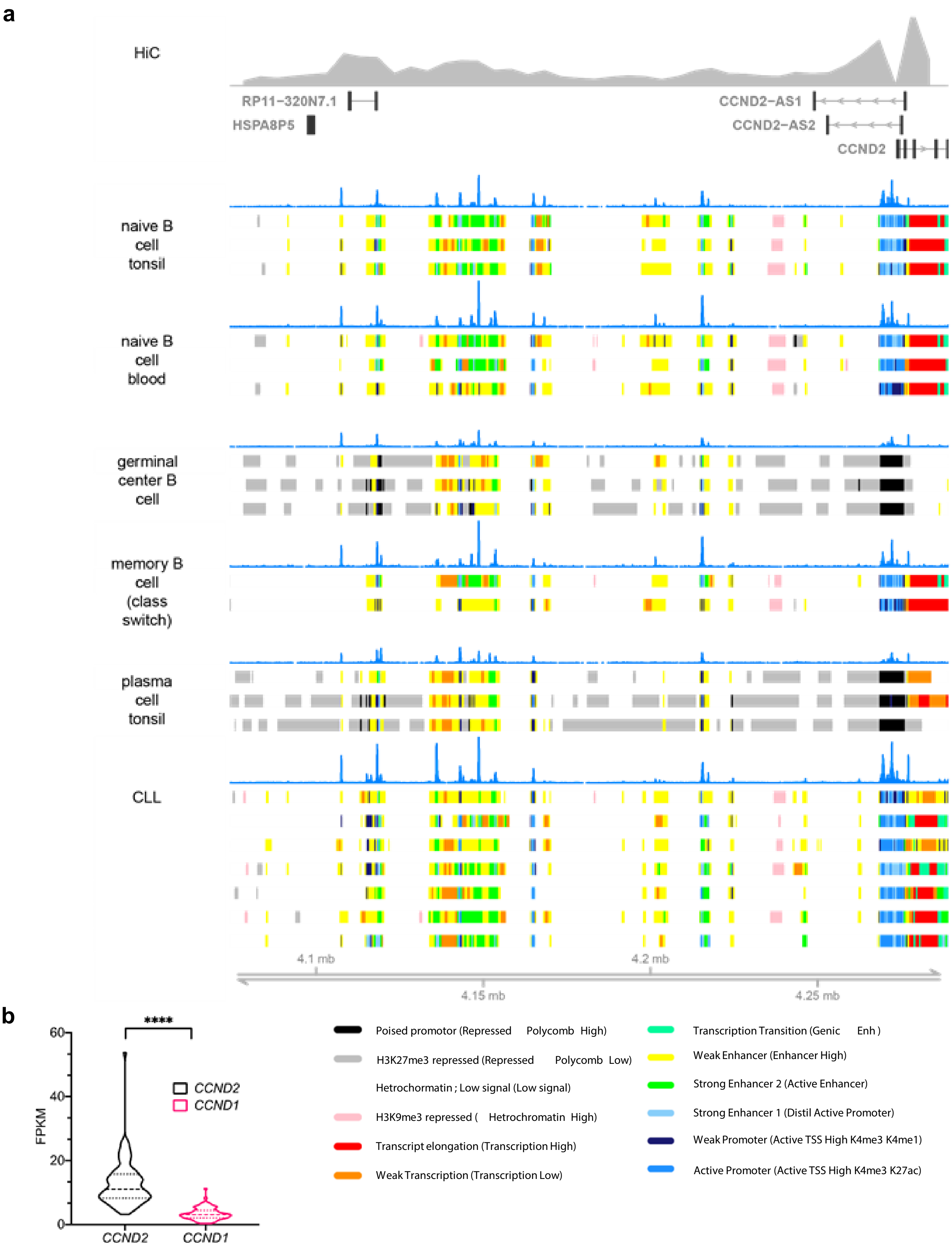
a
HiC
RP11-320N7.1
HSPA8P5
CCND2-AS1
CCND2-AS2
CCND2
naive B cell tonsil
naive B cell blood
germinal center B cell
memory B cell (class switch)
plasma cell tonsil
CLL
4.1 mb
4.2 mb
4.15 mb
4.25 mb
b
****
60
40
20
0
FPKM
CCND2
CCND1
Poised promotor (Repressed Polycomb High)
H3K27me3 repressed (Repressed Polycomb Low)
Hetrochormatin ; Low signal (Low signal)
H3K9me3 repressed (Hetrochromatin High)
Transcript elongation (Transcription High)
Weak Transcription (Transcription Low)
Transcription Transition (Genic Enh)
Weak Enhancer (Enhancer High)
Strong Enhancer 2 (Active Enhancer)
Strong Enhancer 1 (Distil Active Promoter)
Weak Promoter (Active TSS High K4me3 K4me1)
Active Promoter (Active TSS High K4me3 K27ac)

Supplementary Figure 7 (related to Fig 5). Activation of *CCND2* super-enhancer in other haematological malignancies.

a) Chromatin landscape of the *CCND2* locus in chronic lymphocytic leukemia (CLL) and associated normal B cell types from [2]. Colour tracks show ChromHMM states. Key shows Beekman *et al* annotation of chromatin state, with the closest matching state from the model used by Blueprint epigenomics (as shown in Figure 2 and Supp Figure 5). Traces are average ATAC-seq signal over samples of that type. Hi-C track shows Hi-C signal associated with the *CCND2* promoter from GM12878 B cells.

b) RNA-seq signal for *CCND2* and *CCND1* from CLL samples (n=78). Statistical analysis was done by Mann-Whitney test . **** p<0.0001

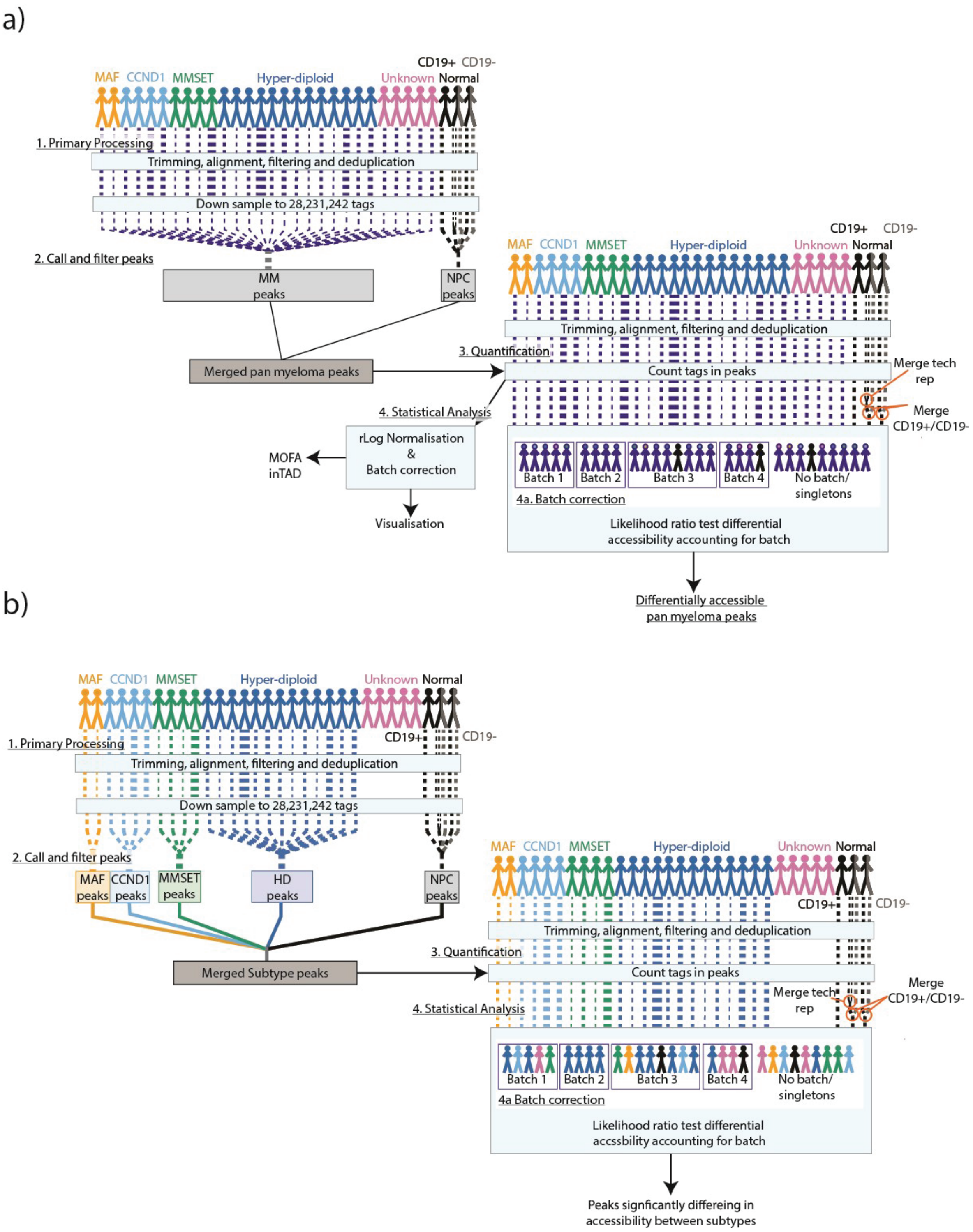

Supplementary Figure 8. Schematic illustration of steps followed for ATAC-seq data analysis in this study. (a) Pan-myeloma analysis; **(b)** subgroup-specific analysis.

2. Supplementary Tables

Supplementary Table 1. List of Antibodies

Antibody	Purpose	Fluorophore Channel	Company/Catalogue Number
**CD138**	positive	FITC	BD Bioscience 552723
**CD319**	positive	PE	eBiosciece 12-2229-42
**CD27**	positive	PECy7	Biolegend 302837
**CD2**<br>**CD3**<br>**CD14**<br>**CD16**<br>**GPA**	negative	Alexa647	Bio-Rad MCA1194A647T<br>Biolegend 300321<br>Biolegend 325611<br>Biolegend 302020<br>Bio-Techne FAB1228R
**CD19**	Pos & Neg	APC efluoro780	eBiosciece 47-0199-42
**CD45**	Positive	Alexa700	Biolegend 368513
**CD38**	positive	Alexa450	eBioscience 48-0389-42
**7AAD**	negative	7AAD	Biolegend 420403
**CD56**	FACs MM	PECy7	eBioscience 25-0567
**CD38**	FACs MM	BV421	BD Bioscience 562445
**MAF**	ChIP		Santa Cruz sc-7866
**H3K27ac**	ChIP		Abcam ab4729
**IgG**	ChIP		Santa Cruz sc-2027

All FACS antibodies have been extensively validated by the manufacturers.
The H3K27Ac ChIP antibody has been extensively validated by the manufacturers.In addition, all ChIP antibodies are validated by ChIP qPCR, against IgG control. In the case of the MAF antibody, cell lines not expressing the protein were used as additional negative controls.

Supplementary Table 2. List of primers and oligos.

Primer	Purpose	Sequence (5'-3')
***CCND2* Peak 1A**	sgRNA	TCTACCTGCCGCACCCACGG
***CCND2* Peak 1B**	sgRNA	CCGTGAGGCCCCTGTGTATC
***CCND2* Peak 2A**	sgRNA	TAGCTGCACTCAGCGCTCGA
***CCND2* Peak 2B**	sgRNA	GCTCAGGCACGACCGTCAGT
***CCND2* Peak 3A**	sgRNA	CAAGATCTGCCACCGTCTGT
***CCND2* Peak 3B**	sgRNA	AGCCTATTAGGGAGAACCCC
***CCND2* Peak 4A**	sgRNA	GCACGTCTATTAAGCGTTGA
***CCND2* Peak 4B**	sgRNA	GACCACGTCGAGAGCTCCCC
***CCND2* Peak PA**	sgRNA	TCTGAAGCGGTGACGCAAGC
***CCND2* Peak PB**	sgRNA	GCTAAATAGGGGGTTTTCGG
Gal4	sgRNA	AACGACTAGTTAGGCGTGTA
***IRF4* sh2**	shRNA (F)	CCGGTTTACTGAAATGCGCTCTTTACTCGAGTAAAGAGCGCATTTCAGTAAATTTTTG
***IRF4* sh2**	shRNA (R)	AATTCAAAAATTTACTGAAATGCGCTCTTTACTCGAGTAAAGAGCGCATTTCAGTAAA
***IRF4* sh3**	shRNA (F)	CCGGGCCATTCCTCTATTCAAGAATCTCGAGATTCTTGAATAGAGGAATGGCTTTTTG
***IRF4* sh3**	shRNA (R)	AATTCAAAAAGCCATTCCTCTATTCAAGAATCTCGAGATTCTTGAATAGAGGAATGGC
***CXXC1* sh1**	shRNA (F)	CCGGGTGTATAATCCTCAGAGCAAACTCGAGTTTGCTCTGAGGATTATACACTTTTTG
***CXXC1* sh1**	shRNA (R)	AATTCAAAAAGTGTATAATCCTCAGAGCAAACTCGAGTTTGCTCTGAGGATTATACAC
***CXXC1* sh3**	shRNA (F)	CCGGCATCCGGATCACTGAGAAGATCTCGAGATCTTCTCAGTGATCCGGATGTTTTTG
***CXXC1* sh3**	shRNA (R)	AATTCAAAAACATCCGGATCACTGAGAAGATCTCGAGATCTTCTCAGTGATCCGGATG
