# Supplementary material for: Chromatin-based, in cis and in trans regulatory rewiring underpins distinct oncogenic transcriptomes in multiple myeloma
Source: Nat Commun. 2021 Sep 14;12:5450. doi: 10.1038/s41467-021-25704-2 (PMC8440555; doi:10.1038/s41467-021-25704-2)
Supplement: Supplementary file 2 — Description of Additional Supplementary Files [file 41467_2021_25704_MOESM2_ESM.docx]

**Description of Additional Supplementary Files**

**File Name:** Supplementary Data 1

**Description:** Clinical information and annotation of primary samples.

**File Name:** Supplementary Data 2

**Description:** Differentially accessible peaks across and within MM subgroups versus normal donor plasma cells.

**File Name:** Supplementary Data 3

**Description:** Differentially expressed genes across and within MM subgroups versus normal donor plasma cells.

**File Name:** Supplementary Data 4

**Description:** Differentially expressed genes within 1Mb of differentially accessible regions per MM subgroup.

**File Name:** Supplementary Data 5

**Description:** Enrichment analysis of differentially expressed genes per MM subgroup.

**File Name:** Supplementary Data 6

**Description:** Epigenomic (chromHMM) states of differentially accessible pan-MM regions across B cell development.

**File Name:** Supplementary Data 7

**Description:** Statistical overview and metrics for transcription factors analysis per MM subgroup, including TF footprinting, dependency scores and network statistics.
