# Supplementary material for: Chromatin-based, in cis and in trans regulatory rewiring underpins distinct oncogenic transcriptomes in multiple myeloma
Source: Nat Commun. 2021 Sep 14;12:5450. doi: 10.1038/s41467-021-25704-2 (PMC8440555; doi:10.1038/s41467-021-25704-2)
Supplement: Supplementary file 10 — Reporting Summary [file 41467_2021_25704_MOESM10_ESM.pdf]

nature research

Corresponding author(s): Anastasios Karadimitris/ Caputo Valentina / Ian Sudbery

Last updated by author(s): 12/03/2021

Reporting Summary

Nature Research wishes to improve the reproducibility of the work that we publish. This form provides structure for consistency and transparency in reporting. For further information on Nature Research policies, see Authors & Referees and the Editorial Policy Checklist .

Statistics

For all statistical analyses, confirm that the following items are present in the figure legend, table legend, main text, or Methods section.

n/a	Confirmed	
☐	☒	The exact sample size (*n*) for each experimental group/condition, given as a discrete number and unit of measurement
☐	☒	A statement on whether measurements were taken from distinct samples or whether the same sample was measured repeatedly
☐	☒	The statistical test(s) used AND whether they are one- or two-sided<br>*Only common tests should be described solely by name; describe more complex techniques in the Methods section.*
☐	☒	A description of all covariates tested
☐	☒	A description of any assumptions or corrections, such as tests of normality and adjustment for multiple comparisons
☐	☒	A full description of the statistical parameters including central tendency (e.g. means) or other basic estimates (e.g. regression coefficient) AND variation (e.g. standard deviation) or associated estimates of uncertainty (e.g. confidence intervals)
☐	☒	For null hypothesis testing, the test statistic (e.g. *F*, *t*, *r*) with confidence intervals, effect sizes, degrees of freedom and *P* value noted<br>*Give P values as exact values whenever suitable.*
☐	☒	For Bayesian analysis, information on the choice of priors and Markov chain Monte Carlo settings
☒	☐	For hierarchical and complex designs, identification of the appropriate level for tests and full reporting of outcomes
☐	☒	Estimates of effect sizes (e.g. Cohen's *d*, Pearson's *r*), indicating how they were calculated

Our web collection on statistics for biologists contains articles on many of the points above.

Software and code

Policy information about availability of computer code

Data collection	High-throughput sequencing data for patient and normal donor BM samples and MM cell lines were generated in-house.<br>Additional MM patient WGS and RNA-seq data were obtained from the MMRF database (https://research.themmrf.org/).<br>The Chromatin State Segmentations (ChromHMM) for 19 B-cell lineage stages we retrieved from The DeepBlue Epigenomic Data Server (https://deepblue.mpi-inf.mpg.de/). CLL samples data were downloaded from http://inb-cg.bsc.es/hcli/IDIBAPS_Biomedical_Epigenomics/CLL_Reference_Epigenome/. Additional information is provided in the Supplementary file.
Data analysis	All computational methods used in this paper are detailed in Supplementary Methods.<br>In short, quality control of High-throughput sequencing data was performed using FastQC (v0.11.3).<br>The human genome Grch38 annotations were obtained from Ensembl (v85).<br>Bowtie2 was used for ChIP-seq data alignment.<br>Picard (4.0.1) was used to mark and remove duplicate reads. MACS2 (2.1) was used for peak calling.<br>Deeptools (v2.0) was used to create signal tracks from bam files.<br>Tools from Homer package (v4.9) were used for motif analysis, super-enhancer calling and annotation of genomic regions against the hg38 human genome, following the default mode.<br>Salmon (v0.11.4) was used to obtain expression estimates and perform fragment GC bias correction.<br>DeSeq2 (v1.18.1) was used for RNA-seq data normalization and differential expression.<br>Batch effects data were removed using limma package (v3.34.9).<br>Unannotated TSS present in samples were obtained by mapping RNA-seq reads using Hisat (v0.1.6).<br>Stringtie (v1.2.3) was used to assemble mapped reads and identify novel transcripts.<br>Cutadapt (v1.9.1) and Sickle (v1.33) were used for ATAC-seq adapters trimming.<br>Functions from the samtools (v1.3.1) and bedtools (v2.20.0) packages were used for file re-formatting and filtering.<br>The R library ‘Annotatr’ (v1.8.0) was used to annotate accessible chromatin regions using the TxDb.Hsapiens.UCSC.hg38.knownGene reference package.

The R library 'Annotatr' (v1.8.0) was used to annotate accessible chromatin regions using the TxDb.Hsapiens.UCSC.hg38.knownGene reference package.
inTAD (v1.2.3) was used to calculate the correlation of gene-expression with peak accessibility in the same TAD.
The R library MOFAtools (v0.99.0) was used for ATAC-seq/RNA-seq combined factor model training and analysis.
Enrichment analyses were carried out using gene sets from either MSigDB (https://www.gsea-msigdb.org/gsea/msigdb/), Enrichr (https://maayanlab.cloud/Enrichr/) or goseq R package (v1.36.0).
The binomial family and logit link R function glm was used for logistic regression analysis. Silhouette scores were calculated with the R package cluster (v2.0.6).
Computation of generalized inverse of the MOFA loadings matrix were performed with the ginv function of pymatlab (v0.2.1) package.
The pyDNAse/Wellington package was used for TF footprinting analysis.
TF motifs were obtained from the HOCOMOCOv1 collection.
TF to gene-target annotation was done with the ChIPpeakAnno R package (v3.8).
Networks visualizations and graph theory analyses were performed using Cytoscape (v3.5).

Custom code used in this work is available at: https://github.com/sudlab/alvarez_et_al .

For manuscripts utilizing custom algorithms or software that are central to the research but not yet described in published literature, software must be made available to editors/reviewers. We strongly encourage code deposition in a community repository (e.g. GitHub). See the Nature Research guidelines for submitting code & software for further information.

Data

Policy information about availability of data

All manuscripts must include a data availability statement. This statement should provide the following information, where applicable:

- Accession codes, unique identifiers, or web links for publicly available datasets
- A list of figures that have associated raw data
- A description of any restrictions on data availability

Raw sequencing data, peak sets and gene quantifications are deposited in GEO Accession GSE153381.
Additional MM patient WGS and RNA-seq data were obtained from the MMRF database (https://research.themmrf.org/).
The Chromatin State Segmentations (ChromHMM) for 19 B-cell lineage stages we retrieved from The DeepBlue Epigenomic Data Server (https://deepblue.mpi-inf.mpg.de/).
Additional data for CLL samples were downloaded from http://inb-cg.bsc.es/hcli/IDIBAPS_Biomedical_Epigenomics/CLL_Reference_Epigenome/.
Further information is provided in the Supplementary file.

Field-specific reporting

Please select the one below that is the best fit for your research. If you are not sure, read the appropriate sections before making your selection.

☒ Life sciences ☐ Behavioural & social sciences ☐ Ecological, evolutionary & environmental sciences

For a reference copy of the document with all sections, see nature.com/documents/nr-reporting-summary-flat.pdf

Life sciences study design

All studies must disclose on these points even when the disclosure is negative.

Sample size	The sample size was defined according to the availability of primary samples and the guidelines of the Ethics Committee. For MM cell line experiments, 2 or 3 biological replicates we performed for each assay. This sample size was sufficient to support significant observations and results described in this study.
Data exclusions	RNA-seq and ATAC-seq data for ND3_CD19neg sample were excluded due to low sequencing quality.
Replication	All primary samples were sufficient for one RNA-seq and ATAC-seq reaction, so replication of primary samples analysis is impossible. after the analysis making replication of the in vivo part of the study impossible. Cell lines data were replicated (other experiments n=2-3). Each replication was successful.
Randomization	Primary samples preparation and analysis was performed randomly, based on the availability of clinical samples. For MM cell line experiments, cells were randomly split into treatment/control groups and subjected to the same culture conditions and processing, to exclude any technical bias.
Blinding	All primary samples were anonymous. Clinical information was only revealed after samples processing , in order to assist in subgroups identification and analysis. For MM cell line experiments, blinding was not relevant as none of the recorded data was subjective (e.g. CRISPRi constructs, GFP+ fluorescence, etc.)

Reporting for specific materials, systems and methods

We require information from authors about some types of materials, experimental systems and methods used in many studies. Here, indicate whether each material, system or method listed is relevant to your study. If you are not sure if a list item applies to your research, read the appropriate section before selecting a response.

Materials & experimental systems		
n/a	Involved in the study	
☐	☒	Antibodies
☐	☒	Eukaryotic cell lines
☒	☐	Palaeontology
☒	☐	Animals and other organisms
☐	☒	Human research participants
☒	☐	Clinical data

Methods		
n/a	Involved in the study	
☐	☒	ChIP-seq
☐	☒	Flow cytometry
☒	☐	MRI-based neuroimaging

Antibodies

Antibodies used	Flow-cytometry: CD138 (BD Bioscience 552723), CD319 (eBiosciece 12-2229-42), CD27 (Biolegend 302837), CD2 (Bio-Rad MCA1194A647T), CD3 (Biolegend 300321), CD14 (Biolegend 325611), CD16 (Biolegend 302020), GPA (Bio-Techne FAB1228R), CD19 (eBiosciece 17-0199), CD45 (Biolegend 368513), CD38 (eBioscience 48-0389-42), 7AAD (Biolegend 420403), CD56 (eBioscience 25-0567), CD38 (BD Bioscience 562445), MAF (Santacruz sc-7866)<br>ChIP-seq: MAF sc-7866 (Santa-Cruz), H3K27Ac ChIP ab4729 (Abcam), IgG ChIP sc-2027 (Santa-Cruz)
Validation	All FACs antibodies have been extensively validated by the manufacturers.<br><br>The H3K27Ac ChIP antibody has been extensively validated by the manufacturers. In addition, all ChIP antibodies are validated by ChIP qPCR, against IgG control. In the case of the MAF antibody, cell lines not expressing the protein were used as additional negative controls.

Eukaryotic cell lines

Policy information about cell lines

Cell line source(s)	MM.1S, U266, KMS12BM , NCI-H929, OPM2, JJN3, HEK-293T (ATCC, Manassas, VA, USA ; DSMZ, Germany)
Authentication	Karyotyping was performed for all cell lines
Mycoplasma contamination	All cell lines were routinely tested (every two weeks) for mycoplasma throughout this study. The cell lines used were confirmed negative.
Commonly misidentified lines<br>(See ICLAC register)	No commonly misidentified lines were used in this study.

Human research participants

Policy information about studies involving human research participants

Population characteristics	The covariate-relevant population characteristics considered in this study were gender, disease sub-category, disease stage and cytogenetic information. Our experimental design considered a roughly equal representation of the covariates characteristics across all processed samples (additional information provided in Suppl.Data1 table).
Recruitment	Samples were processed randomly, based on resources availability. No other inclusion/exclusion criteria were implemented.
Ethics oversight	NHS Health Research Authority, East of England - Cambridge Central Research Ethics Committee Reference: 11/H0308/9

Note that full information on the approval of the study protocol must also be provided in the manuscript.

ChIP-seq

Data deposition

☒ Confirm that both raw and final processed data have been deposited in a public database such as GEO.

☒ Confirm that you have deposited or provided access to graph files (e.g. BED files) for the called peaks.

Data access links
May remain private before publication.

GEO Accession GSE153381

Files in database submission

GSE153381 Chromatin accessibility and gene expression maps of Plasma Cells and Myeloma Plasma Cells Jun 26, 2021

GSE153379 Chromatin accessibility and gene expression maps of Plasma Cells and Myeloma Plasma Cells [ATAC-seq] Jun 26, 2021 TSV TSV

GSM4643219	A17.9	Jun 26, 2021	BED BED BW
GSM4643220	A19.1	Jun 26, 2021	BED BED BW
GSM4643221	A19.2	Jun 26, 2021	BED BED BW
GSM4643222	A19.5	Jun 26, 2021	BED BED BW
GSM4643223	A19.8	Jun 26, 2021	BED BED BW
GSM4643224	A24.7	Jun 26, 2021	BED BED BW
GSM4643225	A19.6	Jun 26, 2021	BED BED BW
GSM4643226	A24.4	Jun 26, 2021	BED BED BW
GSM4643227	A28.13	Jun 26, 2021	BED BED BW
GSM4643228	A28c.14	Jun 26, 2021	BED BED BW
GSM4643229	A26.6B	Jun 26, 2021	BED BED BW
GSM4643230	A26.15B	Jun 26, 2021	BED BED BW
GSM4643231	A27.20	Jun 26, 2021	BED BED BW
GSM4643232	A26.8	Jun 26, 2021	BED BED BW
GSM4643233	A26.9B	Jun 26, 2021	BED BED BW
GSM4643234	A26.10B	Jun 26, 2021	BED BED BW
GSM4643235	A26.12	Jun 26, 2021	BED BED BW
GSM4643236	A26.13	Jun 26, 2021	BED BED BW
GSM4643237	A27.12	Jun 26, 2021	BED BED BW
GSM4643238	A27.18	Jun 26, 2021	BED BED BW
GSM4643239	A26.1	Jun 26, 2021	BED BED BW
GSM4643240	A26.18	Jun 26, 2021	BED BED BW
GSM4643241	A26.19	Jun 26, 2021	BED BED BW
GSM4643242	A26.20	Jun 26, 2021	BED BED BW
GSM4643243	A27.21	Jun 26, 2021	BED BED BW
GSM4643244	A27.22	Jun 26, 2021	BED BED BW
GSM4643245	A24.10	Jun 26, 2021	BED BED BW
GSM4643246	A24.11	Jun 26, 2021	BED BED BW
GSM4643247	A24.8	Jun 26, 2021	BED BED BW
GSM4643248	A17.5	Jun 26, 2021	BED BED BW
GSM4643249	A26.14	Jun 26, 2021	BED BED BW
GSM4643250	A26.11	Jun 26, 2021	BED BED BW
GSM4643251	A27.19	Jun 26, 2021	BED BED BW
GSM4643252	A28.15	Jun 26, 2021	BED BED BW
GSM4643253	A28.7	Jun 26, 2021	BED BED BW
GSM4643254	A28c.3	Jun 26, 2021	BED BED BW
GSM4643255	A28c.5	Jun 26, 2021	BED BED BW
GSM4643256	A28c.6	Jun 26, 2021	BED BED BW
GSM4643257	AJJN3.1	Jun 26, 2021	BED BED BW

GSE153380 Chromatin accessibility and gene expression maps of Plasma Cells and Myeloma Plasma Cells [RNA-seq] Jun 26, 2021 TSV

GSM4643258	RNA-seq_A17.9	Jun 26, 2021
GSM4643259	RNA-seq_A19.1	Jun 26, 2021
GSM4643260	RNA-seq_A19.2	Jun 26, 2021
GSM4643261	RNA-seq_A19.5	Jun 26, 2021
GSM4643262	RNA-seq_A19.8	Jun 26, 2021
GSM4643263	RNA-seq_A24.7	Jun 26, 2021
GSM4643264	RNA-seq_A19.6	Jun 26, 2021
GSM4643265	RNA-seq_A24.4	Jun 26, 2021
GSM4643266	RNA-seq_A28.13	Jun 26, 2021
GSM4643267	RNA-seq_A28c.14	Jun 26, 2021
GSM4643268	RNA-seq_A26.6B	Jun 26, 2021
GSM4643269	RNA-seq_A26.15B	Jun 26, 2021
GSM4643270	RNA-seq_A27.20	Jun 26, 2021
GSM4643271	RNA-seq_A26.8	Jun 26, 2021
GSM4643272	RNA-seq_A26.9B	Jun 26, 2021
GSM4643273	RNA-seq_A26.10B	Jun 26, 2021
GSM4643274	RNA-seq_A26.12	Jun 26, 2021
GSM4643275	RNA-seq_A26.13	Jun 26, 2021

GSM4643276	RNA-seq_A27.12	Jun 26, 2021
GSM4643277	RNA-seq_A27.18	Jun 26, 2021
GSM4643278	RNA-seq_A26.1	Jun 26, 2021
GSM4643279	RNA-seq_A26.18	Jun 26, 2021
GSM4643280	RNA-seq_A26.19	Jun 26, 2021
GSM4643281	RNA-seq_A27.21	Jun 26, 2021
GSM4643282	RNA-seq_A27.22	Jun 26, 2021
GSM4643283	RNA-seq_A24.10	Jun 26, 2021
GSM4643284	RNA-seq_A24.11	Jun 26, 2021
GSM4643285	RNA-seq_A24.8	Jun 26, 2021
GSM4643286	RNA-seq_A17.5	Jun 26, 2021
GSM4643287	RNA-seq_A26.14	Jun 26, 2021
GSM4643288	RNA-seq_A26.11	Jun 26, 2021
GSM4643289	RNA-seq_A27.19	Jun 26, 2021
GSM4643290	RNA-seq_A28.15	Jun 26, 2021
GSM4643291	RNA-seq_A28.7	Jun 26, 2021
GSM4643292	RNA-seq_A28c.3	Jun 26, 2021
GSM4643293	RNA-seq_A28c.5	Jun 26, 2021
GSM4643294	RNA-seq_A28c.6	Jun 26, 2021
GSM4643295	RNA-seq_AJJN3.1	Jun 26, 2021

Genome browser session
(e.g. UCSC)

NA

Methodology

Replicates

No replicates could be performed for primary samples assays. ChIPseq against MAF was performed for n=2 biogical replicates.

Sequencing depth

At least 40M reads were used for each experiments

Antibodies

MAF ChIP sc-7866
H3K27Ac ChIP ab4729
IgG ChIP sc-2027

Peak calling parameters

All analyses details are reported in the supplementary file.

Data quality

All analyses details are reported in the supplementary file.

Software

All analyses details are reported in the supplementary file.

Flow Cytometry

Plots

Confirm that:

- [x] The axis labels state the marker and fluorochrome used (e.g. CD4-FITC).
- [x] The axis scales are clearly visible. Include numbers along axes only for bottom left plot of group (a 'group' is an analysis of identical markers).
- [x] All plots are contour plots with outliers or pseudocolor plots.
- [x] A numerical value for number of cells or percentage (with statistics) is provided.

Methodology

Sample preparation

Patient and normal donor BM samples
BM aspirates were obtained after written informed consent and research ethics committee approval (Research Ethics Committee reference: 11/H0308/9). Patient BM aspirates were subjected to CD138 immunomagnetic bead selection. Post-selection purity was assessed by flow-cytometry.

Normal donor BM mononuclear cells were negatively selected for T cells and monocytes by sequential immunomagnetic bead selection (CD3 and CD14-EasySep StemCell Technologies). CD19+ and CD19- plasma cells were defined as CD138+CD319+CD27+CD45+CD38+ CD2-CD3-CD14-CD16-GPA- and flow-sorted after staining with corresponding mAbs and 7AAD (FACSAriaII, BD Biosciences).
All samples were processed immediately after selection for ATAC-seq and RNA-seq.

ATAC-seq, RNA-seq and ChIP-seq
ATAC-seq was performed as described 35. RNA-seq was performed on extracted RNA by preparing directional libraries according to Illumina (New England Biolabs) instructions, as described 36.For ChIP-seq, 10x106 cells were fixed in 1% formaldehyde. After quenching, washing and lysis in ChIP buffer, chromatin was sonicated, followed by immunoprecipitation using an anti-MAF Ab, washed and de-crosslinked. Immunoprecipitated and input DNA were extracted and used for library preparation using NEBNext Ultra II DNA Library Prep kit from Illumina.
Next generation sequencing was performed on an Illumina HiSeq 4000 platform.

CRISPRi
Upon lentiviral transduction, the inducible sgRNA-CRISPR-dCas9-KRABv2-GFP-puro vector was introduced into the cells of interest. Ten days after puromycin selection, cells were induced with doxycycline for 4 days, FACS-sorted based on GFP marker and their CCND2 expression was assessed by RT-qPCR.

Additional details can be found in the Supplementary file.

Instrument

Real-time qPCR was performed using a StepOne Plus Real-Time PCR (Applied Biosystems) instrument. ATAC-seq, ChIP-seq and RNA-seq libraries were sequenced with an Illumina HiSeq 4000 platform. Bioruptor UCD-200 (Diagenode) was used for sonication. Virus ultra-centrifugation was performed with theThermo Sorvall Ultracentrifuge, MTX (Applied Biosciences). Flow-cytometry analysis was performed on a BD LSR Fortessa Analyser. Cell sorting was done with a MA900 Multi-Application Cell Sorter (Sony Biotechnology) or an AriaII FACS sorter (BD Biosciences).

Software

All computational methods used in this paper are detailed in Supplementary Methods.
In short, quality control of High-throughput sequencing data was performed using FastQC (v0.11.3).
The human genome Grch38 annotations were obtained from Ensembl (v85).
Bowtie2 was used for ChIP-seq data alignment.
Picard (4.0.1) was used to mark and remove duplicate reads. MACS2 (2.1) was used for peak calling.
Deeptools (v2.0) was used to create signal tracks from bam files.
Tools from Homer package (v4.9) were used for motif analysis, super-enhancer calling and annotation of genomic regions against the hg38 human genome, following the default mode.
Salmon (v0.11.4) was used to obtain expression estimates and perform fragment GC bias correction.
DeSeq2 (v1.18.1) was used for RNA-seq data normalization and differential expression.
Batch effects data were removed using limma package (v3.34.9).
Unannotated TSS present in samples were obtained by mapping RNA-seq reads using Hisat (v0.1.6).
Stringtie (v1.2.3) was used to assemble mapped reads and identify novel transcripts.
Cutadapt (v1.9.1) and Sickle (v1.33) were used for ATAC-seq adapters trimming.
Functions from the samtools (v1.3.1) and bedtools (v2.20.0) packages were used for file re-formatting and filtering.
The R library 'Annotatr' (v1.8.0) was used to annotate accessible chromatin regions using the TxDb.Hsapiens.UCSC.hg38.knownGene reference package.
The R library 'Annotatr' (v1.8.0) was used to annotate accessible chromatin regions using the TxDb.Hsapiens.UCSC.hg38.knownGene reference package.
inTAD (v1.2.3) was used to calculate the correlation of gene-expression with peak accessibility in the same TAD.
The R library MOFAtools (v0.99.0) was used for ATAC-seq/RNA-seq combined factor model training and analysis.
Enrichment analyses were carried out using gene sets from either MSigDB (https://www.gsea-msigdb.org/gsea/msigdb/), Enrichr (https://maayanlab.cloud/Enrichr/) or goseq R package (v1.36.0).
The binomial family and logit link R function glm was used for logistic regression analysis. Silhouette scores were calculated with the R package cluster (v2.0.6).
Computation of generalized inverse of the MOFA loadings matrix were performed with the ginv function of pymatlab (v0.2.1) package.
The pyDNAse/Wellington package was used for TF footprinting analysis.
TF motifs were obtained from the HOCOMOCOv1 collection.
TF to gene-target annotation was done with the ChIPpeakAnno R package (v3.8).
Networks visualizations and graph theory analyses were performed using Cytoscape (v3.5).

Custom code used in this work is available at: https://github.com/sudlab/alvarez_et_al .

Cell population abundance

Approximately 50,000 cells were used for ATAC-seq. RNA extraction was performed on ~100,000 cells (for qPCR or RNA-seq analysis). ChIP-seq was performed on 3-5*10^7 cells. For transduction experiments, ~50-100*10^3 cells were used per condition.

Gating strategy

Multiple myeloma plasma cells were purified by two rounds of CD138 immunomagnetic selection. The purity pre- and post-selection was assessed using the following markers: CD138(+), CD45(+), CD19(-/+), CD56(+) and CD38(+) markers.

Normal donor samples were stained and sorted for CD138+, CD319+, CD27+, CD45+ and CD38+ (positive), for CD2-, CD3-, CD14-, CD16-, GPA- and 7AAD- (negative) and CD19+/- (positive or negative).

☒ Tick this box to confirm that a figure exemplifying the gating strategy is provided in the Supplementary Information.
